# Probiotic disruption of quorum sensing reduces virulence and increases cefoxitin sensitivity in methicillin-resistant *Staphylococcus aureus*

**DOI:** 10.1038/s41598-023-31474-2

**Published:** 2023-03-16

**Authors:** Monica Angela Cella, Thomas Coulson, Samantha MacEachern, Sara Badr, Ali Ahmadi, Mahdis Sadat Tabatabaei, Alain Labbe, Mansel William Griffiths

**Affiliations:** 1 Department of Mechanical Engineering, École de Technologie Supérieure (ÉTS), Montreal, QC H3C 1K3 Canada; 2 MicroSintesis Inc., Victoria, PE COA 2G0 Canada; 3 Canadian Research Institute for Food Safety, University of Guelph, Guelph, ON N1G 2W1 Canada; 4 Food Science Department, University of Guelph, Guelph, ON N1G 2W1 Canada

**Keywords:** Applied microbiology, Bacteriology

## Abstract

Therapies which target quorum sensing (QS) systems that regulate virulence in methicillin-resistant *Staphylococcus aureus* (MRSA) are a promising alternative to antibiotics. QS systems play a crucial in the regulation of MRSA antibiotic resistance, exotoxin production, antioxidant protection and immune cell evasion, and are therefore attractive therapeutic targets to reduce the virulence of a pathogen. In the present work the the effects of bioactive peptides isolated from two strains of lactic acid bacteria were tested against antibiotic resistance, carotenoid production, resistance to oxidative killing and biofilm structure in two clinical MRSA isolates. The results obtained from fractional-inhibitory concentration assays with bulk and semi-purified bioactive molecules showed a significant synergistic effect increasing cefoxitin mediated killing of MRSA. This was coupled to a six-fold decrease of the major membrane pigment staphyloxanthin, and a 99% increase in susceptibility to oxidative stress mediated killing. Real-time quantitative PCR analysis of the QS-genes *agrA* and *luxS*, showed differential expression between MRSA strains, and a significant downregulation of the hemolysin gene *hla.* Light microscopy and scanning electron microscopy revealed alteration in biofilm formation and clustering behavior. These results demonstrate that bioactive metabolites may be effectively applied in tandem with beta-lactam antibiotics to sensitize MRSA to cefoxitin. Moreover, these results shown that several key QS-controlled virulence mechanisms are diminished by probiotic metabolites.

## Introduction

Overuse of antibiotics has historically contributed to the rise of antibiotic resistant bacteria by selecting for emerging resistance^1^, and it is also no longer expected that dependence on antibiotic monotherapies will be able to keep pace against emerging resistant strains of bacteria^2,3^. One strategy to help manage the spread of antimicrobial resistance is to lower the selective pressure that contributes to emerging resistance by limiting the use of established antimicrobials^4^. Consequently, there is a greater focus in researching alternatives to traditional monotherapies of antibiotic drugs^3,5^. Particularly, alternative therapeutics that interfere with bacterial quorum sensing (QS) and cell-to-cell communication are a promising approach to better manage the spread of antimicrobial resistance. Bacterial QS systems use highly sophisticated regulatory mechanisms to aid bacterial pathogenesis by adapting the expression of virulence genes to the fluctuating environment of the host during infection. At low pathogen densities, signaling molecules used to communicate between cells are low in concentration, but accumulate to a threshold concentration as the cell population density increases. Once the threshold is reached, the QS signalling molecules are taken up by the cell via specific transporters and transcriptional regulators are activated which in turn induce the virulence genes required at that stage of pathogenesis^6^. QS systems are not directly involved in essential metabolic processes and, theoretically at least, these non-killing alternatives would place less selective pressure on pathogens with the capability to express resistance^7,8^. Moreover, as QS systems often play a vital role in typical characteristics and behaviours of bacteria that contribute to their overall virulence, such as toxin production and immune cell evasion, QS disruptors are intriguing therapeutic candidates in the fight against bacterial infection and sepsis^9,10^.

One such novel alternative that has recently emerged as a contender to combat pathogenic bacteria is the use of probiotic bacteria and their metabolites that act as QS disruptors^8^. Although scientific evidence to support claims of the benefits of probiotics on human and animal health is scarce, recent work has elucidated several mechanisms by which probiotics act to directly interfere with pathogen virulence. Metabolic peptides produced by *Lactobacillus acidophilus* have been shown to reduce virulence in pathogenic bacterial strains by reducing their use of QS to sense and communicate in their environment: a crucial contributor to the pathogen’s ability to produce toxins, invade host cells, evade immune cells, and form biofilms^11,12^. Metabolites isolated from the cell free spent medium (CFSM) of *Bifidobacterium* cultures have also been shown to down regulate the main regulatory genes controlling the virulence factors necessary for attachment and adhesion in *Salmonella enterica* serovar Typhimurium and enterohaemorrhagic *Escherichia coli* O157:H7^13,14^. Notably, it has been shown in vivo that the probiotic *Bacillus subtilis* produces quorum quenching metabolites that significantly contributed to the exclusion of the commensal pathogen *Staphylococcus aureus* in the gut within rural Thai populations^15^.

*Staphylococcus aureus* is a gram-positive pathogen of serious concern. Gram-positive bacteria utilize autoinducing oligopeptides in intercellular QS-controlled gene expression systems, and communication via these impermeable autoinducers is mediated by specialized receptors. The accessory gene regulator (*agr*) pathway is one of the most well-described QS systems in *S. aureus*, comprising a two-component receptor histidine kinase (AgrC) and its transcriptional response regulator (AgrA)^16^. The *agr* system is influenced by cell population density and regulates virulence expression as required by *S. aureus* in the various stages of infection^17^. It has also been shown that AgrA possesses an oxidation-sensing ability that is critical in *S. aureus* defense against oxidative stress and immune cell evasion^18^, and that the *agr* system is essential in density-dependent regulation of exotoxins such as alpha hemolysin and cell surface protein expression^19,20^. Previous research has also shown that the *agr* system displays a significant regulatory effect on the *mecA* gene that controls methicillin resistance in highly virulent community-acquired methicillin resistant *S. aureus* (MRSA)^21–23^. Thus, it has been proposed that the *agr* system should be considered as a significant candidate for QS disruption and novel therapeutic intervention in *S. aureus*^9^. A second QS-regulated system is the alternative sigma factor B (*sigB*), which modulates the stress response systems of many Gram-positive bacteria including *S. aureus.* SigB also regulates virulence traits such as carotenoid biosynthesis and antibiotic resistance mechanisms^24^. Furthermore, deletion of *sigB* in a MRSA strain led to increased susceptibility to cell-wall active antibiotics such as methicillin and oxacillin^25^. Thus, an intriguing idea is the application of non-antibiotic QS disrupting molecules in combination with established antibiotics as a novel combination therapy.

It is proposed that QS disruptors produced by probiotic bacteria could be utilized in combating antimicrobial resistance by aiding ineffectual antibiotics to return to more efficacious states against certain MRSA strains. Additionally, due to the overarching regulatory presence of multiple QS systems in MRSA, the disruption of QS-regulated factors that greatly contribute to MRSA virulence (e.g. oxidant survival) may also be achieved by these probiotic metabolites. To analyze the potential capabilities of probiotic compounds in virulence reduction, the cell-free spent media (CFSM) of the probiotic lactic acid bacteria (LAB) *Enterococcus faecium* NCIMB 30616 (“Ef 30616”) was selected. A second probiotic strain of lactic acid bacteria, *Lactococcus lactis* ATCC 11454 (“Ll 11454”), was also investigated to determine if the effect was conserved amongst many probiotic species. Moreover, two clinical isolate strains of MRSA were selected as targets to compare the reduction several key virulence factors well described in highly virulent MRSA populations^20^: staphyloxanthin and carotenoid synthesis^23^, alpha hemolysin production, increased hydrogen peroxide survival and resistance to oxidant killing^23,26^, possession of the mobile genetic element *Staphylococcal* cassette chromosome (SCC) containing the genes for antibiotic resistance machinery and regulation and expression of the *mecA* gene located on the SCC^9,17,21,22,27^.

## Results

### Minimum inhibitory concentration testing

**Figure 1:**
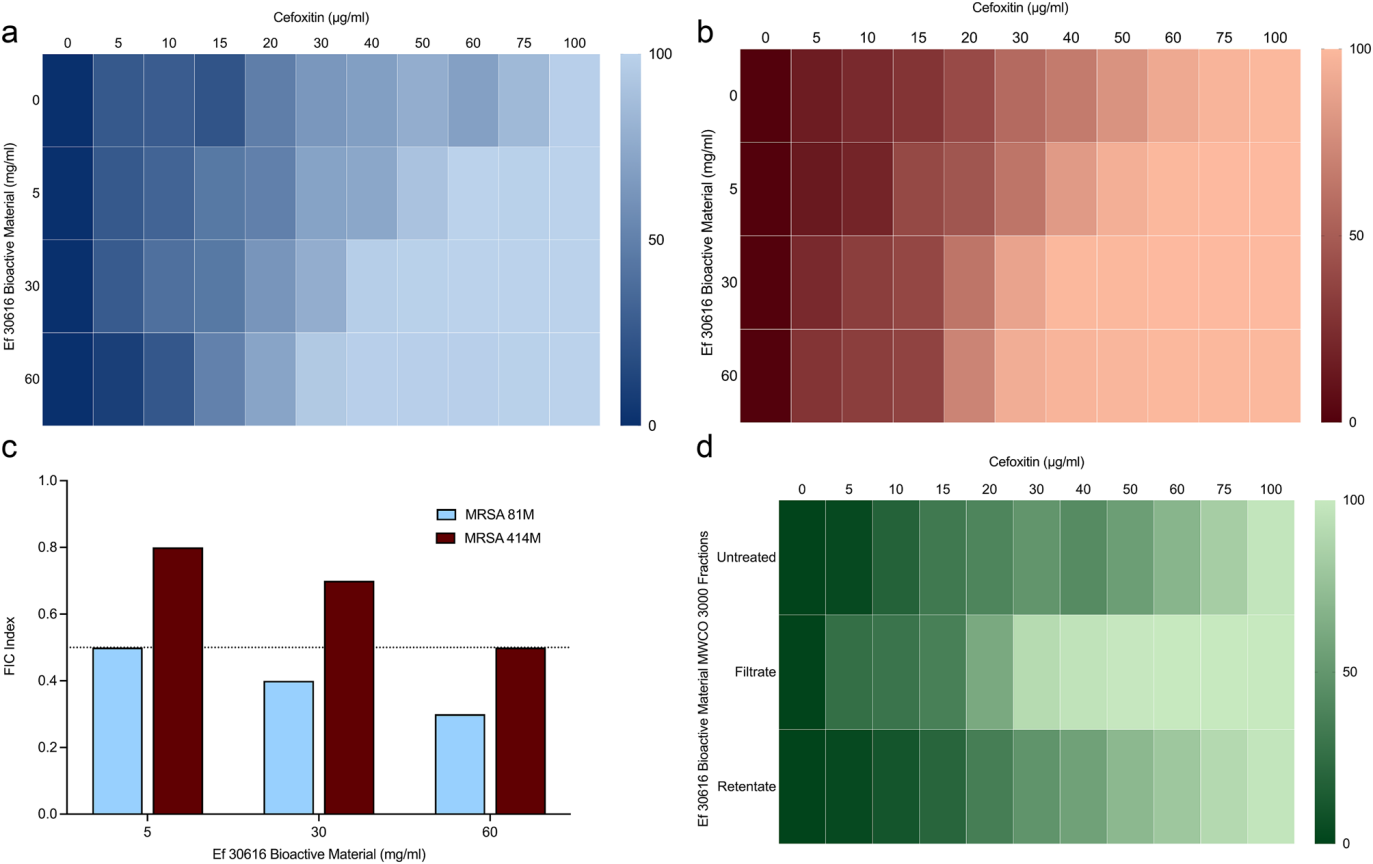
Heat plots of MIC percent growth inhibition of the two representative clinical MRSA strains by combinational testing of the beta-lactam antibiotic cefoxitin (0–100 µg/mL) and Ef 30616 bioactive metabolites (0, 5, 30 and 60 mg/mL): (**a**) MIC heat plots of MRSA 81M and (**b**) MIC heat plots of MRSA 414M. (**c**) FIC index values for the combinatory effects of EfMSI1 bioactive metabolites and cefoxitin for MRSA strains 81M and 414M. (**d**) Heat plot of the percent growth inhibition of MRSA 81M for the combination testing of cefoxitin (0–100 µg/mL) and Ef 30616 bioactive metabolites (30 mg/mL) MWCO 3000 filtrate (i.e. < 3000 Da) and retentate (i.e. > 3000 Da) (n = 3). Averages of biological replicates are shown for heat maps and are presented as the means in terms of percent growth inhibition (ANOVA, *p* < 0.05 Dunnett’s multiple comparison test).

To investigate the effects of probiotic material, the individual minimum inhibitory concentrations (MIC) of two clinical MRSA strains, strain 81M (“MRSA 81M”) and strain 414M (“MRSA 414M”) were first determined against the beta-lactam antibiotic cefoxitin, and which resulted in cefoxitin MICs of 100 µg/mL and 60 µg/mL for each strain, respectively. Cefoxitin was selected for this study as this antibiotic has been shown to strongly upregulate the SSC *mecA* (PBP2a) pathway for antibiotic resistance in MRSA strains^28^. The addition of three concentrations of filter-sterilized Ef 30616 bulk CFSM containing bioactive metabolites (5, 30 and 60 mg/mL) were added in checkerboard fashion to a range of cefoxitin concentrations (0–100 µg/mL) with equal starting inocula of each respective MRSA strain. The results indicate a concentration-dependent decrease in the minimum concentration of cefoxitin required to reduce the MRSA cell density by more than 90% when incubated with the bioactive metabolites for 24 h (Fig. 1a,b). The fractional inhibitory concentration (FIC) index was used to evaluate the potency of combining the non-antimicrobial bioactive compound with cefoxitin^29^. The FIC is a mathematical expression used to describe the effects of combinations of two or more antibiotics or of an antibiotic and a non-antibiotic compound; effects may be described as antagonistic, indifferent, additive or synergistic as defined by the FIC index^30^. The criteria index for determining the results of the FIC calculations between cefoxitin and the Ef 30616 bioactive material was implemented as follows: a synergistic outcome was observed if the combination exceeded the observed additive effects of the individual components (FIC ≤ 0.5), an additive outcome was observed at the sum of the effects of the individual components (FIC > 0.5–1.0), an indifferent outcome was observed if the combination was equal to the effect observed from the most active component (FIC > 1.0–2.0), and an antagonistic outcome was observed when the combination of the two compounds had a reduced effect in comparison to the most active individual component (FIC > 2.0). The FICs obtained for both MRSA 81M and 414M show an inverse relation for FIC values with increasing concentration of Ef 30616 bioactive metabolites (Fig. 1c). Only strain 81M showed synergistic FIC values at all concentrations of CFSM tested, with a significant decrease in MIC at both 30 and 60 mg/ml relative to the untreated CFSM control (*p* < 0.0001, Dunnett’s multiple comparison test). However, strain 414M only had additive effects and did not lead to significantly different MIC with any concentration of CFSM used. Therefore, the two strains of MRSA in this study have differential sensitivity to the bioactive material when treated with cefoxitin, indicating strain specific differences in response to CFSM and/or antibiotic resistance.

Following the determination of the FIC values, MRSA 81M was selected for further MIC testing as it has greater sensitivity to the bioactive material (see above) and showed synergistic FIC values at all tested concentrations whereas MRSA 414M did not express a similar pattern. The concentration of 30 mg/mL bioactive material was selected as it was the lowest concentration that had a FIC value well under 0.5. The Ef 30616 bioactive material was split into two fractions using a 3000 dalton (Da) molecular weight cut-off (MWCO) centrifugal filter, with the first fraction containing only the filtrate (< 3000 Da) and the second fraction containing only the washed and resuspended retentate (> 3000 Da). A checkerboard method was again used to determine the MIC of the MRSA 81M strain using the same combination of cefoxitin concentrations (0–100 µg/mL) with equal starting inoculum, and a similar pattern in reduced MIC was seen in the filtrate (Fig. 1d).

## Size-exclusion chromatography FIC testing

It was necessary to confirm that the reduction in MRSA 81M MIC values seen with both bulk and fractioned CFSM were due to bioactive peptides, and not inhibitory molecules produced by LAB such as organic acids. Preparative size exclusion chromatography (SEC-FPLC) was used to separate the bulk CFSM into fractions containing specific metabolites, with small peptides eluting into fraction four (“peptide fraction”). Each of the SEC fractions were resuspended to a concentration equivalent to 5, 15 or 30 mg/mL of the original CFSM and tested in a checkerboard titration with cefoxitin to determine the effect on MRSA 81M MICs. If one or more of the fractions are enriched for bioactive metabolites, a strong additive or synergistic effects should be seen even with low concentrations of the material.

**Figure 2:**
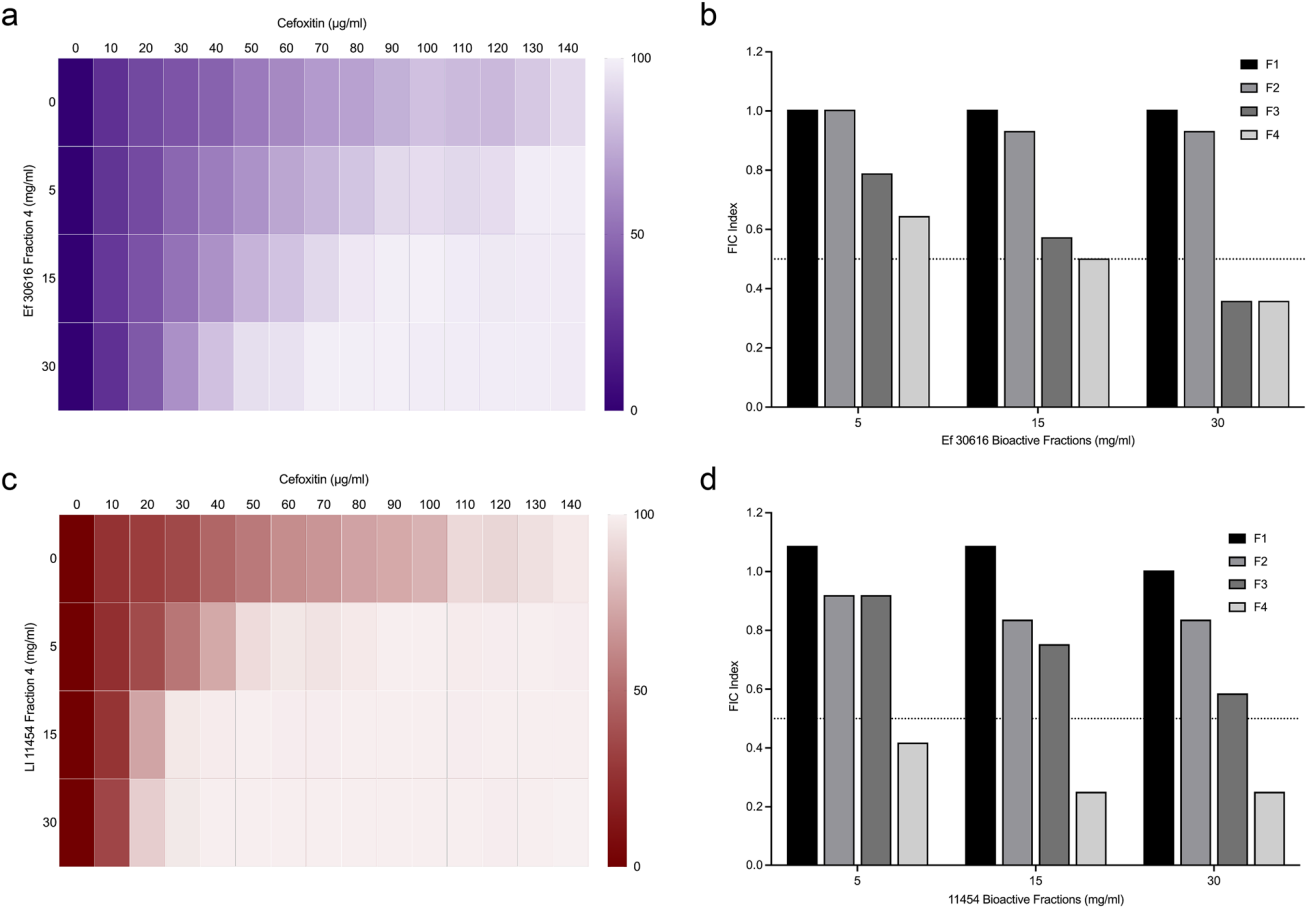
Heat plots of MIC percent growth inhibition of MRSA 81M in combination with cefoxitin (0–140 µg/mL) and SEC fraction four from (**a**) Ef 30616 or (**b**) Ll 11454 at 5, 15 and 30 mg/mL of equivalent bulk material. (**c**) FIC index values for Ef 30616 fractions one through four and (**d**) Ll 11454 fractions one through four. Averages of biological replicates are shown for heat maps and are presented as the means in terms of percent growth inhibition (ANOVA, *p* < 0.05 Dunnett’s multiple comparison test).

The results indicate that Ef 30616 fraction four was able to reduce MRSA 81M MICs in a concentration-depended manner (Fig. 2a). FIC index values were synergistic at 15 and 30 mg/mL of material, and additive at 5 mg/mL (Fig. 2b). As this fraction contains no organic acids, it may be concluded that the effects are due to the small bioactive peptides it contains. Fraction three was also found to decrease 81M MIC values, however this was only synergistic at the highest concentration used (Fig. 2b, Supplementary Fig. S1). Neither fractions one nor two had any effect on bacterial MIC (Supplementary Fig. S1), further confirming that small peptides are the bioactive molecules responsible for decreasing MRSA 81M resistance to cefoxitin.

Fractionated bioactive material from a second probiotic bacteria, *L. lacti*s 11454, was also tested against MRSA 81M in a FIC assay and the peptide fraction was also found to reduce MIC values (Fig. 2c). There were synergistic effects of Ll 11454 peptides and cefoxitin at all concentrations tested, resulting in a significant decrease in MIC relative to the untreated control (Fig. 2d) (*p* < 0.0001, Dunnett’s multiple comparison test). Interestingly, despite Ef 30616 fraction four containing less proteinaceous material then the equivalent Ll 11454 fraction, the Ef 30616 material still significantly reduces MRSA 81M growth in cefoxitin, suggesting that the peptides responsible for the effect is potent at low concentrations and constitute only a small portion of the total protein in the material. From these results it is demonstrated that small bioactive peptides resulting from probiotic fermentation can reduce MRSA antibiotic resistance.

## Small colony variants and hemolysis

**Figure 3:**
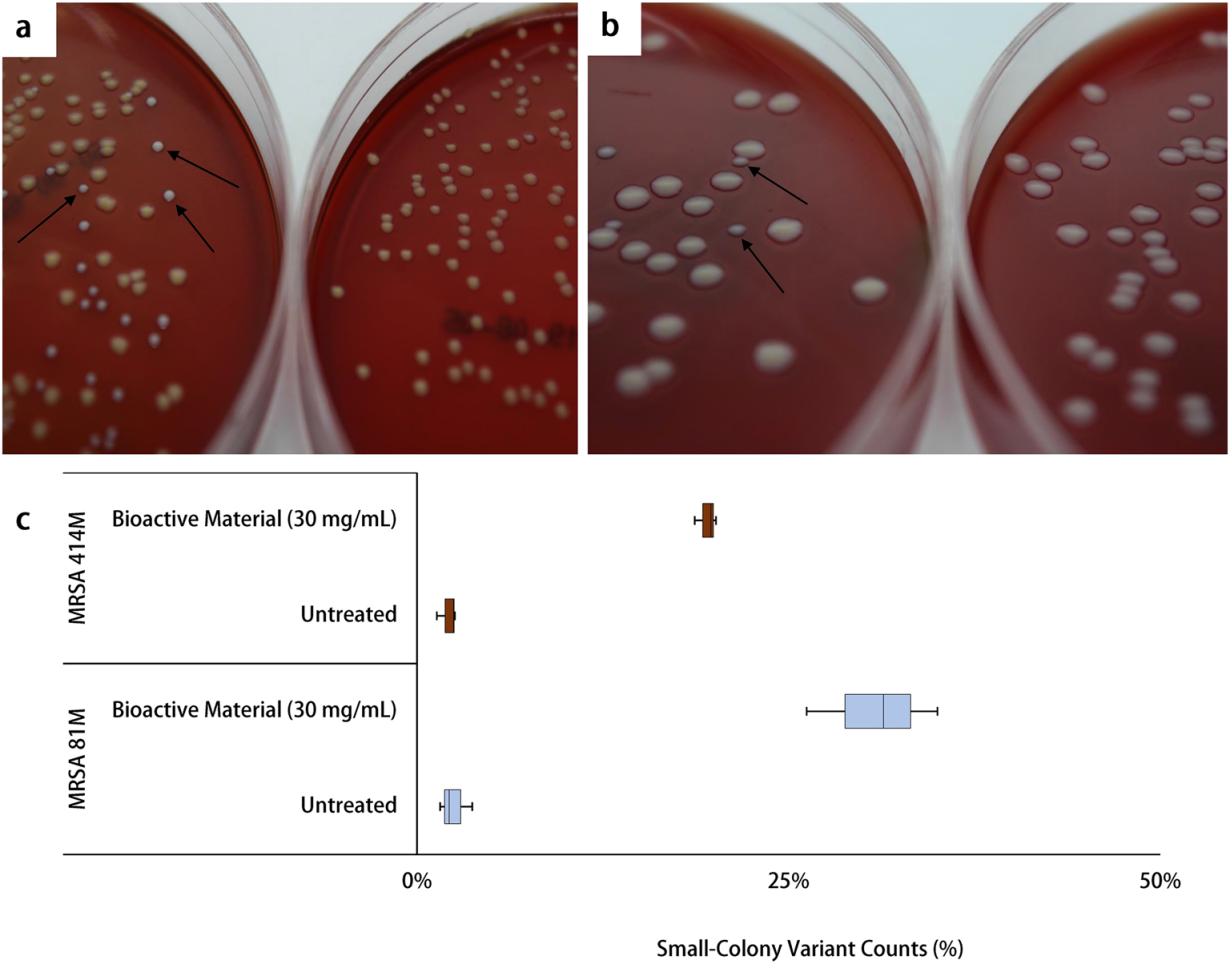
(**a**) Colonies of MRSA 81M growing on blood agar after treatment with 30 mg/mL Ef 30616 bioactive material (left) or no treatment (right) and (**b**) colonies of MRSA 414M growing on blood agar after treatment with 30 mg/ml Ef 30616 bioactive material (left) or no treatment (right). Arrows indicate SCVs lacking carotenoid pigmentation and reduced hemolysis. (**c**) Percentage of SCV to wild-type colonies for MRSA 81M (n = 3) and MRSA 414M (n = 3) following 24 h incubation at 37 +/− 1 °C with and without Ef 30616 bioactive material (30 mg/mL) (U = 0; *p* < 0.05; Mann–Whitney). Central black bars indicate the mean, and the upper and lower whiskers how the maximum and minimum, respectively.

Populations of small colony variants (SCVs) may arise in *S. aureus* cultures when cells are exposed to environmental stressors such as antibiotics^31^. Such SCVs were observed when bioactive treated cultures of MRSA were plated on blood agar plates. Following incubation for 24 h at 37 ± 1 °C, plating of bioactive-treated MRSA 81M revealed small white colonies with minimal to no hemolysis rings visible; comparatively, the untreated 81M wildtype colonies were mostly uniform and maintained their distinct orange coloration and hemolytic phenotype (Fig. 3a,b). A total of 30.9% of MRSA 81M colonies had completely lost their orange pigment and appeared as SCVs. A similar pattern was seen for MRSA 414M, however, only 19.5% of total colonies counted had lost their pigmentation (Fig. 3c). The SCV phenotype was retained when the original bioactive treated SCV was re-streaked and grown on blood agar plates (Supplementary Fig. S2). All controls were negative for growth or contamination on the blood agar.

## Carotenoid measurements and antioxidant susceptibility

**Figure 4:**
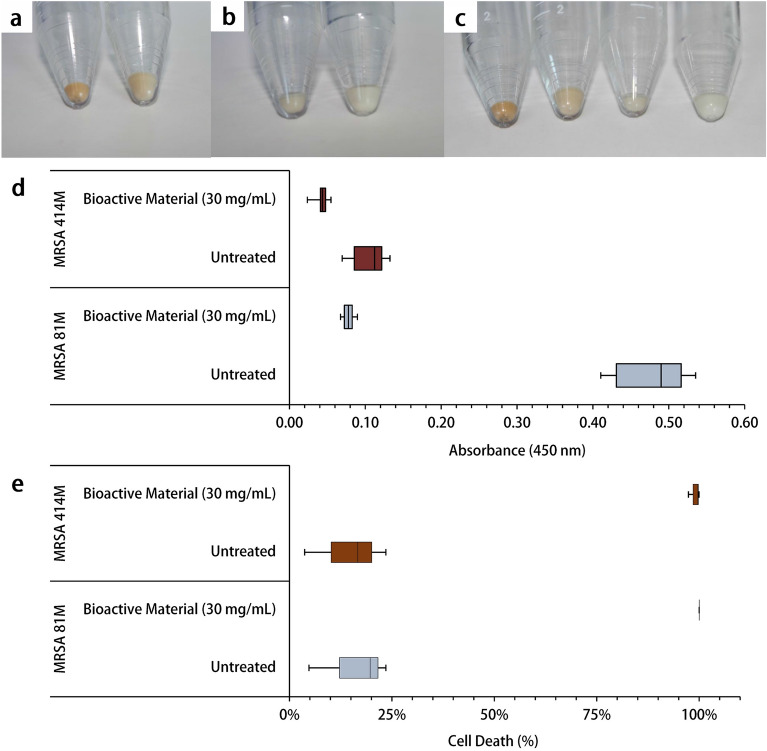
MRSA cell pellets lack carotenoid pigmentation following 24 h incubation with Ef 30616 bioactive material (30 mg/mL) at 37 °C ± 1 °C: (**a**) untreated MRSA 81M (left) and bioactive-treated MRSA 81M (right) pellets, (**b**) untreated MRSA 414M (left) and bioactive-treated MRSA 414M (right) pellets, and (**c**) comparison of all four pellets (from left to right) untreated MRSA 81M, bioactive-treated MRSA 81M, untreated MRSA 414M, and bioactive-treated MRSA 414M. (**d**) Carotenoid absorbance measurement at 450 nm (A450) from each of the two treatments described for both strains (n = 3) (U = 0; *p* < 0.05; Mann–Whitney). (**e**) Percent decrease of *Staphylococcal* survival to 1.5% v/v hydrogen peroxide for MRSA 81M (n = 3) and MRSA 414M (n = 3) following 1 h incubation at 37 °C ± 1 °C in PBS solution with and without Ef 30616 bioactive treatment (30 mg/mL) (W = 0; *p* < 0.05; Wilcoxon signed-rank). Central black bars indicate median, and the upper and lower whisker caps show the maximum and minimum, respectively, for box plots (**d**) and (**e**).

It was observed that following 24 h incubation at 37 °C ± 1 °C with the Ef 30616 bioactive metabolites, strain 81M cells showed significant overall reduction in orange coloration (Fig. 4a); while MRSA 414M exhibited very little pigmentation (Fig. 4b,c). Quantification of carotenoids by methanol extraction and absorbance measurements at 450 nm (A450) corroborated the visual observation as there was a significant six-fold difference in carotenoid concentration as measured by absorbance between treatments of the 81M cells, but less than three-fold difference in carotenoid between treatments of the MRSA 414M cells (U = 0; *p* < 0.05; Mann–Whitney) (Fig. 4d). With reduced carotenoid synthesis, it was hypothesized that the Ef 30616 bioactive metabolites would increase the sensitivity of the MRSA strains to oxidant killing. Hydrogen peroxide was added at 1.5% v/v to untreated and bioactive-treated cultures of each respective MRSA strain and incubated at 37 °C ± 1 °C for 1 h in PBS solution; cell counts (CFU/mL) were determined both before and after the 1 h incubation (Supplementary Fig. S3). The bioactive-treated cells showed 99.67% and > 99.99% cell death for MRSA strains 414M and 81M, respectively (W = 0; *p* < 0.05; Wilcoxon signed-rank); as opposed to the untreated 414M and 81M cells, which exhibited only 16.67% and 19.70% cell death, respectively (Fig. 4e). However, the reduction in untreated 414M was not deemed significant (W = 5; *p* > 0.05; Wilcoxon signed-rank).

## Regulation of quorum sensing and virulence gene expression

**Figure 5:**
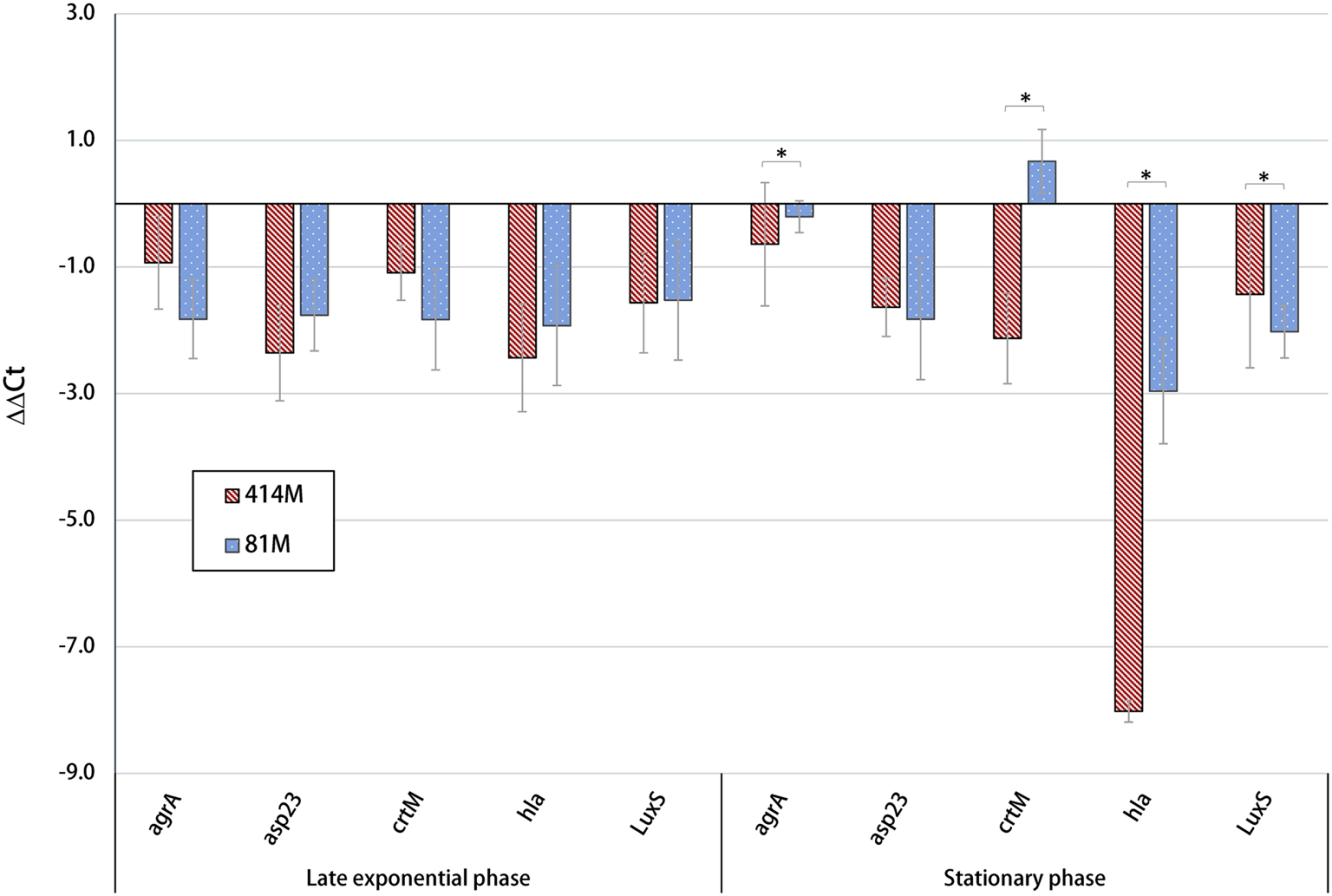
Differential expression of MRSA virulence-related genes in relation to untreated controls of MRSA strains 81M and 414M following incubation with Ef 30616 bioactive material (30 mg/mL) at 37 °C ± 1 °C; cultures were incubated to the late exponential phase and stationary phase. The 16s rRNA was used as a housekeeping gene. Data are shown as averages and bars indicate the standard deviation (n = 3). Statistically significant differences between strains indicated by asterisks (ANOVA; *p* < 0.05).

Analysis of relative mRNA expression was performed to indicate the relationship between QS and virulence gene expression potentially impacted by the Ef 30616 bioactive material (Fig. 5). As many QS-related systems, including *sigB*, have been shown to be more highly activated at late exponential growth stages^24^, expression was monitored for the selected genes at late exponential and early stationary phase for the respective strains. The accessory gene regulator (Agr) QS system did not show significant differential expression relative to the untreated control for the transcriptional response regulator *argA*. The gene *asp*23 was monitored as an indicator of SigB activity, as previously described^32^ and the results indicate that the Ef 30616 probiotic metabolites mildly interfere with key genes related to quorum sensing via SigB in MRSA strains 414M and 81M.

Expression of *hla*, encoding alpha-hemolysin, was strongly downregulated in stationary phase, particularly in strain 414M. This further verifies that bioactive metabolites are reducing hemolysis, as was seen with SCVs on blood agar plates. Interestingly, *crtM*, which encodes dehydrosqualene synthase responsible for catalyzing the first step in staphyloxanthin biosynthesis, seemed to have slight up regulation in strain 81M during early stationary phase, while 414M had a very low level of down regulation. The qPCR results demonstrate differences in virulence gene expression between the two strains of MRSA in response to treatment with bioactive material.

## Scanning electron and light microscopy

**Figure 6:**
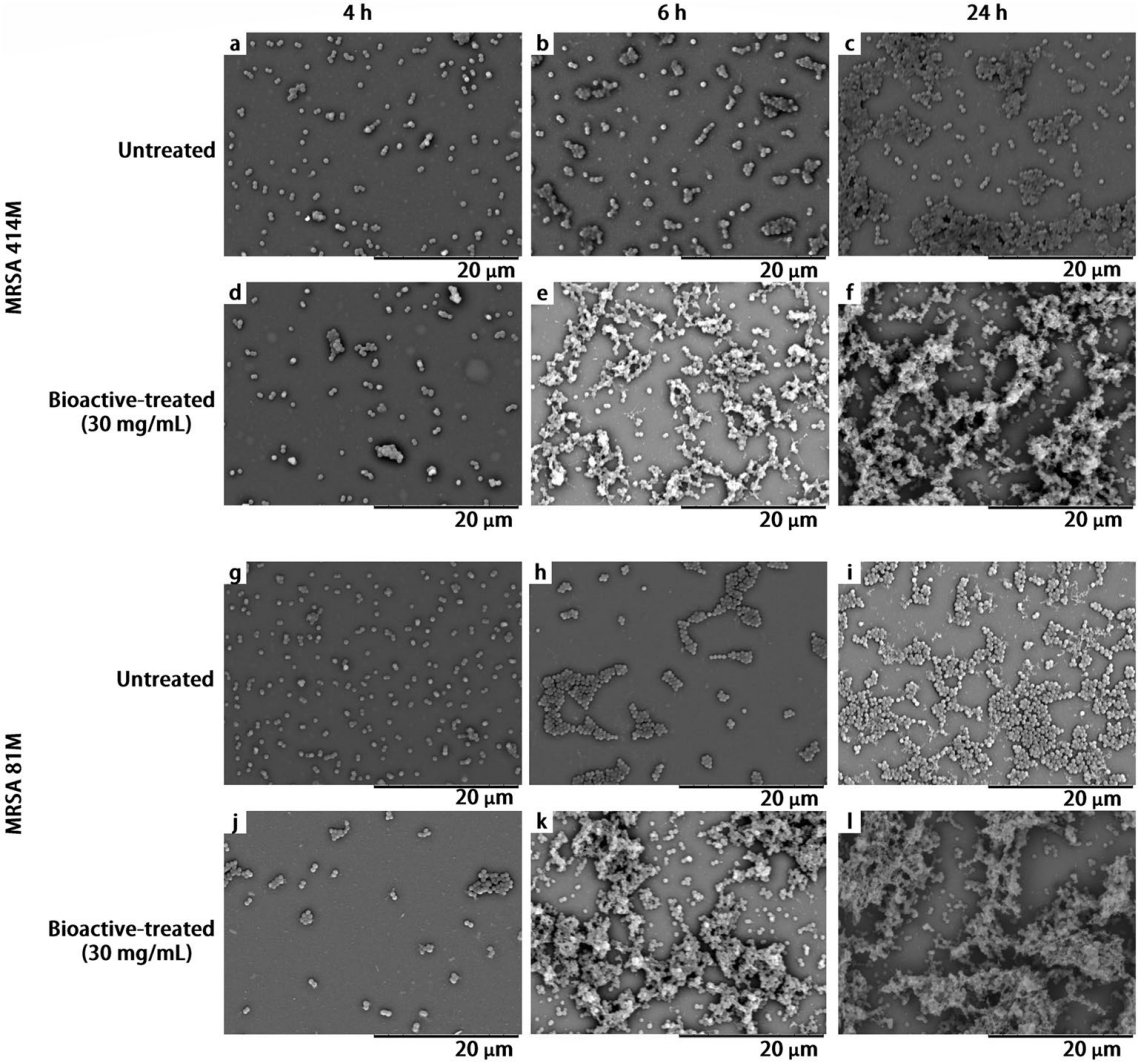
Scanning electron microscopy indicating the progression of biofilm formation of both untreated and bioactive-treated MRSA 81M and 414M cultures incubated over a 24 h period at 37 °C ± 1 °C and imaged at the following intervals: untreated MRSA 414M images at (**a**) 4 h, (**b**) 6 h and (**c**) 24 h, Ef 30616 bioactive-treated MRSA 414M at (**d**) 4 h, (**e**) 6 h and (**f**) 24 h, untreated MRSA 81M at (**g**) 4 h, (**h**) 6 h and (**i**) 24 h, and Ef 30616 bioactive-treated MRSA 81 M at (**j**) 4 h, (**k**) 6 h and (**l**) 24 h. Magnification = 4000×. Scale bar = 20 µm.

**Figure 7:**
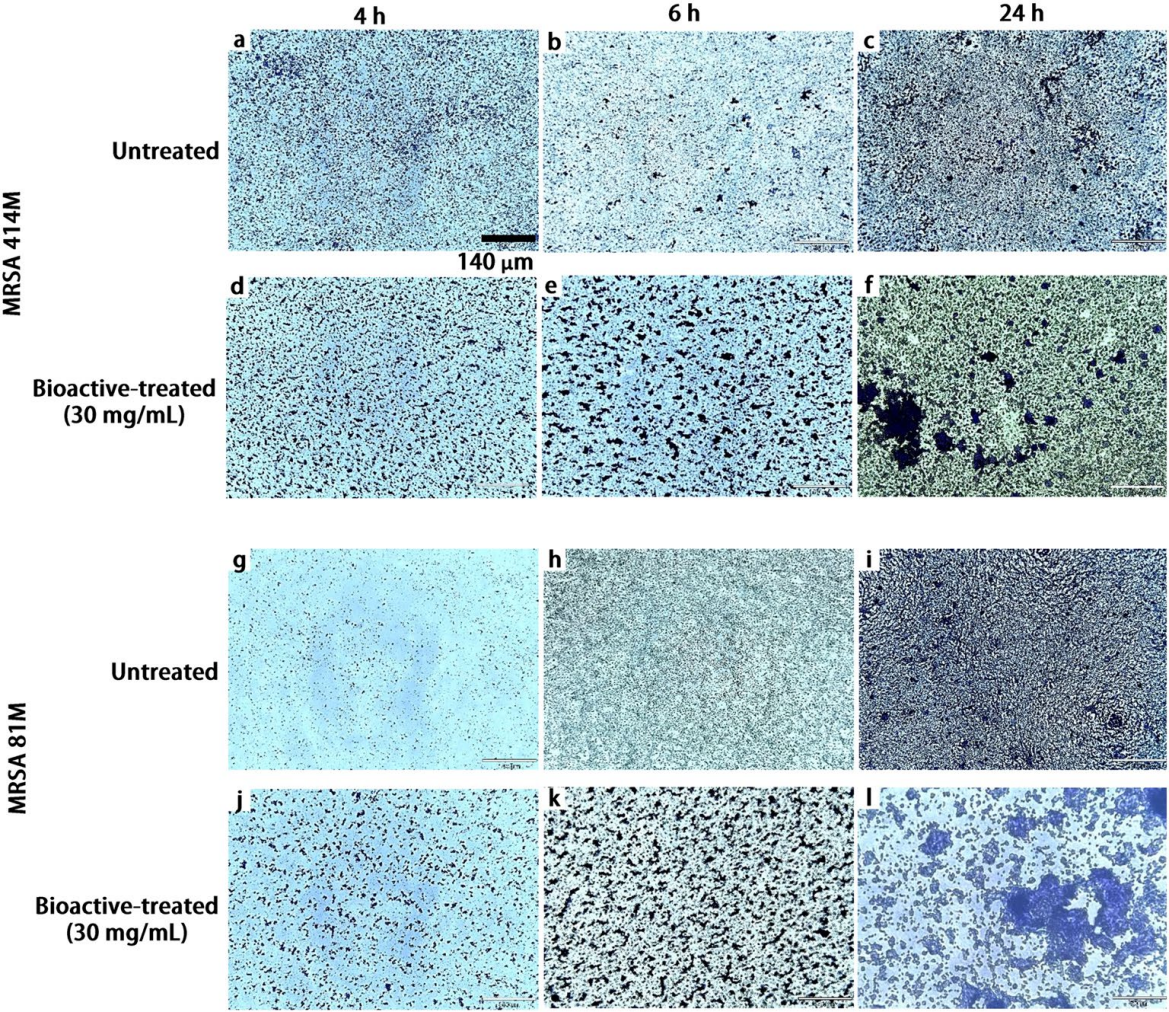
Light microscopy images of different growth stages of both untreated and Ef 30616 bioactive-treated MRSA 81M and 414M cultures incubated over a 24 h period at 37 °C ± 1 °C and imaged at the following intervals: untreated MRSA 414M at (**a**) 4 h, (**b**) 6 h and (**c**) 24 h, bioactive-treated MRSA 414M at (**d**) 4 h, (**e**) 6 h and (**f**) 24 h, untreated MRSA 81M at (**g**) 4 h, (**h**) 6 h and (**i**) 24 h, and Ef 30616 bioactive-treated MRSA 81M at (**j**) 4 h, (**k**) 6 h and (**l**) 24 h. Objective = 10×. Scale bar = 140 µm.

MRSA 81M and 414M strains incubated with and without 30 mg/mL of the probiotic metabolites were evaluated using light microscopy and scanning electron microscopy (SEM) following, 4, 6 and 24 h incubation at 37 °C ± 1 °C. SEM imaging showed that both bioactive-treated MRSA 81M and 414M strains started to aggregate at 6 h incubation and form progressively multilayered structures (Fig. 6d–f, j–l), relative to untreated cells (Fig. 6a–c, g–i). Light microscopy further showed that the bioactive-treated MRSA cells began to gather and form more prominent clusters (Fig. 7d–f, j–l). In contrast, the untreated MRSA cells formed typical grape-like clusters without forming multilayered clusters (Fig. 7a–c, g–i). Moreover, treatment with the bioactive compound did not appear to affect the cell shape or size: both untreated and bioactive-treated MRSA 81M and 414M individual cells appeared as coccoid.

## Discussion

The present study demonstrates that metabolites isolated from the CFSM of *E. faecium* strain 30616 and *L. lactis* 11454 increase the sensitivity of two clinical MRSA strains to cefoxitin, a beta-lactam antibiotic that is no longer clinically suitable against MRSA infections. As cefoxitin works by impeding the cross-linking of peptidoglycan layers within the bacterial cell wall, the impact of the probiotic bioactive metabolites on cell wall components is synergistically beneficial to cefoxitin’s mechanism of action. Our data indicate that bioactive peptides from *E. faecium* Ef 30616 and *L. lactis* 11454 can act as non-antimicrobial adjuvants in conjunction with an antibiotic, and critically, may have the potential to “re-sensitize” MRSA to no longer clinically relevant antibiotics such as cefoxitin. Furthermore, our results also indicate that these Ef 30616 bioactives can impede virulence traits that support MRSA infections: resistance to oxygen species, staphyloxanthin biosynthesis as well as hemolysin production mediated by QS systems in *S. aureus.* As carotenoid pigments greatly contribute to *Staphylococcal* fitness, particularly in immune cell evasion, the inhibition of carotenoid synthesis could be a potential target for therapeutic intervention in *S. aureus* infections^27^. Lastly, the appearance of greater concentrations of SVCs within the overall MRSA population may indicate a shift in MRSA cells with reduced expression of key QS-related genes within the population during later stages of growth. However, in terms of biofilm-related *S. aureus* infections, implementation of probiotic QS-disrupting compounds may not be beneficial as QS-interruption has shown to provide advantages in selection of SVCs and overall biofilm formation^6,33^.

The FIC values for MRSA 81M indicated that there was a synergistic combinatory effect for all three concentrations of Ef 30616 bioactive material, whereas the FIC values for MRSA 414M only showed a synergistic effect at the highest concentration used. These results indicate a strain-specific differences in sensitivity to the antibiotic for the two MRSA strains. Bioactive- only control wells showed no negative effects on MRSA growth for both strains, indicating that the mechanism of action of the bioactive metabolites is not inherently antimicrobial against MRSA, and that MRSA growth inhibition was only observed when the Ef 30616 bioactive metabolites were combined with cefoxitin. The MIC results of the bioactive fractions clearly show that the filtrate contained the greatest activity and reduced the MIC of cefoxitin; however, some residual activity was detected in the retentate, which resulted in the MIC being slightly less than the untreated control. Moreover, two washes of the retentate that were performed also showed some residual bioactive activity (Supplementary Fig. S4). These results indicate that the majority of Ef 30616 compounds containing bioactivity against the two MRSA clinical strains tested were approximately ≤ 3000 Da in size.

By separating the bulk material into chemically defined fractions, bioactive peptides were demonstrated to be responsible for the observed reduction in MRSA strain 81M antibiotic resistance to cefoxitin. The bulk CFSM and MWCO filtrates still contain many media components such as residual sugars, unhydrolyzed proteins as well as organic acids resulting from fermentation. Lactic acid bacteria are known to inhibit the growth of *S. aureus* through acidification of the environment^34^, and it was therefore essential to remove organic acids which may otherwise impact *S. aureus* susceptibility to cefoxitin. Despite there being much less material in the Ef 30616 peptide fraction compared to Ll 11454, the effect was still strong enough to cause a synergistic reduction in FIC values. This suggests that these proteobiotic molecules may act with extremely high potencies, working at very low concentrations. Similar results were observed in a study by Kou et al.^35^, wherein the authors found that as little as 1 µg/mL of a synthetic quorum quenching molecule were able to reduce MRSA MICs to the early-generation beta-lactam antibiotics nafcillin and cephalothin by 50-fold. Furthermore, these molecules were demonstrated to be effective at reducing death when applied in combination with cephalothin in a mouse MRSA bacteraemia/sepsis model^36^. Artificial peptides have also been employed to enhance the potency of early generation antibiotics through disruption of the cell envelope^37^. Our results clearly demonstrate that the natural fermentation processes of lactic acid bacteria may be harnessed to generate bioactive peptides which possess potent anti-virulence activity.

The golden pigment displayed by antioxidant carotenoids was significantly reduced for MRSA 81M and MRSA 414M following treatment with 30 mg/mL of Ef 30616 bioactive metabolites. Remarkably, although the wildtype MRSA 414M possessed very little orange pigmentation originally, treatment with the Ef 30616 bioactive material caused the MRSA 414M to lose most of its pigment resulting in an almost purely white pellet. The change in cell pellet color following 24 h incubation with the Ef 30616 bioactive metabolites is indicative of probiotic disruption of proper cell wall structuring: carotenoids are also thought to be involved in stabilizing the *S. aureus* membrane during infection and pathogenesis and maintaining cell rigidity against host defenses^26,38^. Moreover, these surface-accumulated compounds contribute to the overall virulence of *S. aureus* by providing protection against photosensitization and desiccation, as well as by acting as potent antioxidants that quench toxic reactive oxygen species that are used by immune cells^39^. The reduction in characteristic yellow-orange coloring of colloquially known “golden staph” MRSA is likely due to the disruption of QS-related two component systems that are reported to be essential to hydrogen peroxide tolerance in *S. aureus*, including the alternate sigma factor *sigB*. Moreover, although the cell pellets were nearly identical in total CFU/mL counts between the untreated control and the bioactive-treated MRSA cells following 24 h incubation, the cell pellets were less dense and formed larger cell pellets (Fig. 4a–c) which appears to be a result of the advanced biofilm formation following incubation of the MRSA with the bioactive CFSM metabolites, as revealed by microscopy imaging (Figs. 6, 7). Loss of membrane components such as staphyloxanthin affect membrane integrity, leading to changes in aggregation behavior and biofilm formation. It is interesting to note that, although the two bioactive-treated MRSA strains showed advanced progression in aggregation and biofilm formation, they both exhibited increased susceptibility to cefoxitin over 24 h indicating that the aggregation and biofilm production did not aid the MRSA cells in replicating in the presence of the cefoxitin. These finding are of critical interest as sub-inhibitory concentrations of beta-lactam antibiotics may induce biofilm formation in MRSA, and that these results demonstrate that bioactive peptides allow for increased susceptibility of MRSA to antibiotics even during biofilm formation.

Interestingly, following incubation with 1.5% v/v hydrogen peroxide, both bioactive-treated MRSA 81M and MRSA 414M showed significant reduction in cell death at > 99.99% and 99.67%, respectively, even though lingering orange pigment appeared to remain in the bioactive-treated MRSA. However, this low pigment level was possibly from intermediate pigment 4,4′-diaponeurosporene, which has been indicated previously through *sigB* interruption^40^. As wildtype MRSA 414M appeared to have little carotenoid production (compared to MRSA 81M), an alternative theory is that the bioactive metabolites are also able to disrupt the intrinsic component of the *agr* system responsible for sensing oxidative environments^8^. In terms of toxin production, the transcription of *hla* varies amongst strains of MRSA^41^, and downregulation by the bioactive material may be through a conserved mechanism like quorum sensing. The reduction in *hla* was phenotypically observed in the growth of MRSA colonies following treatment with the bioactive material, which showed strong reduction in hemolysis rings on blood agar media for both strains. In addition, most of the colonies with reduced hemolysis displayed different morphologies than the wildtype MRSA: colonies were small, slow-growing and had lost most of their original pigmentation (Fig. 3a,b). These colonies have been previously described in MRSA cultures as small colony variants. Disruption of the *agr* system has been linked to SVCs within wildtype MRSA populations, and which have been shown to display reduced virulence and increased cell aggregation. Together, these findings support the notion that certain Ef 30616 proteobiotic molecules may disrupt typical cell wall synthesis in MRSA by blocking carotenoid production that acts as a crucial antioxidant, resulting in increased sensitivity to oxidant killing.

## Conclusions

These results demonstrate that naturally generated quorum-quenching molecules have the potential to combat the rise of multi-drug antibiotic resistant bacteria. It is possible that through proteobiotic-mediated disruption of MRSA cell-wall structures such as staphyloxanthin, the efficacy of the host immune response could be increased as the bacterial cells become more suspectable to macrophage mediated killing through production of reactive oxygen species. Although these results agree with our previous findings regarding the QS-disrupting activity of the probiotic metabolites found within the CFSM of other probiotic LAB, further research is required to elucidate the complete mechanism of action of these bioactive metabolites. Identifying how these molecules impede quorum sensing control of virulence will also have important implications in their application in a clinical setting to target a bacterial infection. However, adjuvant technologies developed from probiotic bacteria that interrupt *S. aureus* QS systems are potentially serious contenders in combating MRSA infections.

## Methods

### Probiotic bacterial strain

A frozen glycerol stock culture of *Enterococcus faecium* 30616 (“Ef 30616”) (previously identified as *Lactobacillus acidophilus* strain DSM 13241, redesignated after MALDI-TOF microbial identification) was obtained from the Canadian Research Institute for Food Safety (University of Guelph, Ontario, Canada). This stock probiotic strain was used to produce bioactive materials containing probiotic metabolites for all experiments used in this study. A second probiotic strain, *Lactococcus lactis* 11454 (“Ll 11454”) was obtained from the American Type Culture Collection (ATCC) and used to produce bioactive containing material for MIC tests.

### Staphylococcal bacterial strains

Two clinical isolates of *S. aureus* bacteria, MRSA 81M SPA t008 (“MRSA 81M”) and MRSA 414M SPA t034 (“MRSA 414M”), were obtained from the Atlantic Veterinary College (AVC) at the University of Prince Edward Island (Charlottetown, PE, Canada). The presence of methicillin resistance via the SCC *mecA* gene pathway in both strains was confirmed with oxacillin disk diffusion and *mecA* gene primers (Supplementary Table S1)^42^. The clinical isolates were maintained on tryptic soy agar (TSA) plates supplemented with 5% v/v sterile defibrinated sheep blood (Cedarlane, Burlington, Ontario, Canada). MRSA cultures were grown in BBL™ cation-adjusted Mueller Hinton media (Becton, Dickinson and Company, Sparks, MD, USA) for all experimental assays.

### Preparation of probiotic bioactive material

*E. faecium* Ef 30616 was inoculated in 200 mL of 0.22 µm filter-sterilized modified De Man, Rogosa and Sharpe (MRS) medium (VWR International, Mississauga, Ontario, Canada). This bottle culture was incubated without shaking at 37 ± 1 °C for 18 h. The culture was then used to inoculate 4 L of chemically defined medium (CDM) with 200 g whey protein isolate (Canadian Protein) and 25 g lactose. The vessel culture was incubated statically at 37 ± 1 °C for 48 h. Following incubation, the Ef 30616 cells were isolated from the liquid phase by centrifugation at 12,000× *g* for 30 min at 4 °C (Avanti J-20 XPI, Beckman Coulter, Canada). The cell-free supernatant containing the probiotic metabolites was then frozen at − 80 °C and freeze dried. The dried Ef 30616 cell-free supernatant was kept in long-term storage in powder form at − 20 °C until needed.

### Resuspension of probiotic bioactive material

Dried supernatant was weighed out and resuspended in cation-adjusted Mueller Hinton media at the desired concentration (5, 30 and 60 mg/mL). The pH of the resuspended material was checked with an Accumet^®^ AE150 pH meter (Fisher Scientific, Waltham, USA) and adjusted as needed to a pH of 7.3 ± 0.1. The resuspended solution was then centrifuged at 5000× *g* for 15 min at room temperature with a Centrifuge 5804 (Eppendorf, Hamburg, Germany). The remaining liquid was separated from the debris pellet and filtered through a Supor^®^-800 0.45 µm membrane filter (Pall Corporation, New York, USA) with a 40/35 Synthware vacuum filtration apparatus (Kemtech America, Los Angeles, USA) to remove remaining colloidal material. The filtrate was then filter-sterilized using a Basix™ 25 mm 0.2 µm syringe filter (Fisher Scientific, Waltham, USA). The samples were stored at − 20 °C until needed.

### Preparation of molecular weight cut off (MWCO 3000) fractions

Amicon^®^ Ultra 15 mL centrifugal filters with a Molecular Weight Cut Off (MWCO) of 3000 Da (Millipore Sigma, Burlington, USA) were used for ultrafiltration of the resuspended probiotic cell-free supernatant. Following resuspension as described above, 10 mL of the cell-free supernatant was added to the MWCO 3000 centrifugal filter tube and centrifuged at 5000 rpm for 1 h. The MWCO 3000 filtrate was collected; the remaining retentate fraction was collected by rinsing the filter head twice with 10 mL of cation-adjusted Mueller Hinton media for each rinse. Following the two rinses, an additional 10 mL of media was used to resuspend and collect the retentate. All collected fractions were then filter-sterilized with a 0.2 µm syringe filter to remove any contamination. The MWCO 3000 liquid retentate and filtrate solutions were stored at − 20 °C until needed for the experiments.

### Preparation of size-exclusion chromatography (SEC) fractions

Dried supernatant was resuspended in Milli-Q water to a concentration of 50 mg/mL and sonicated for 15 min to improve solubilization. Following resuspension, the samples were centrifuged for 20 min at 8000× *g* and the supernatant filter sterilized with a 0.22 µm syringe filter. Samples were separated on a XK 26/100 preparative column packed with Superdex 30 and run using a AKTA Explorer (Cytiva Life Sciences, Marlborough, USA). Material was eluted with 10% acetonitrile and 0.1% trifluoracetic acid at a flow rate of 3 mL/min. The elution curve was monitored by measuring the pH and absorbance at 280 and 214 nm. The desired elution’s were collected and pooled into defined fractions then frozen at − 80 °C followed by freeze drying. Lyophilized material was stored at − 80 °C until needed.

### Preparation of cefoxitin for MIC testing

The antibiotic selected for minimum inhibitory concentration (MIC) testing in the two MRSA strains was cefoxitin as outlined in the Clinical and Laboratory Standards Institute (CLSI) guidelines for MIC testing of *Staphylococcal* species^43^. Cefoxitin in powder form (Alfa Aesar, Haverhill, USA) was weighed with an analytical balance and resuspended in methanol at 10 mg/mL and stored at − 20 °C until needed.

### Minimum inhibitory concentration (MIC) testing

MIC testing was performed with respect to the Clinical and Laboratory Standards Institute (CLSI) guidelines for MIC testing of *Staphylococcal* species^43^. Overnight cultures of each respective clinical MRSA strain were diluted 1000-fold to obtain approximately 10^6^ CFU/mL as the starting inoculate. Dilution ranges for cefoxitin were from 0 to 100 μg/mL. Clinical MRSA strain MICs with cefoxitin were determined by broth microdilution with cefoxitin in cation-adjusted Mueller Hinton media in Costar^®^ clear 96-well standard flat-bottom microplates (Corning^®^ Inc., Corning, USA). Media-only aliquots were added as sterility checks and were used as background control, while the 100 µg/mL cefoxitin well served as a positive control of antibiotic-mediated killing. All test sample volumes were 200 µL per well with duplicates. Following sample preparation and aliquoting, the MIC test plates were sealed with parafilm and incubated at 35 ± 1 °C and 200 rpm shaking for 24 h in a VWR^®^ Incubating Mini Shaker and temperature was monitored with a Fisherbrand™ digital thermometer (Fisher Scientific, Waltham, USA). Following incubation, the plates were cooled to room temperature and turbidity was measured using a SpectraMax M2 microplate reader (Molecular Devices, San Jose, USA) at a wavelength of 600 nm. The lowest concentrations of cefoxitin that resulted in either 90% or greater reduction in turbidity compared to the respective positive-growth controls were defined as the MIC. All MIC tests were performed in three biological replicates. Following MIC checkerboard testing, as a clinical strain with a high MIC, MRSA 81M was selected for testing of ultrafiltration and SEC fractions of the bioactive material to better elucidate the bioactives’ sizes and chemical identity. The MWCO 3000 and SEC fraction MIC tests were performed identically to the MIC test method outlined above.

### Fractional inhibitory concentration (FIC) index

Checkerboard MIC analyses were performed for three pre-determined probiotic bioactive material concentrations to determine the synergistic, additive, or antagonistic combinatory effects with cefoxitin against the clinical MRSA strains. Dilution ranges of the bioactive material were 5, 30 and 60 mg/mL of freeze dried Ef 30616 cell-free supernatant, or 5, 15 and 30 mg/mL of freeze dried Ef 30616 and Ll 11454 SEC fractions. Dilution ranges for cefoxitin used in FIC testing were 0–100 µg/mL for bulk material, and 0–140 µg/mL for SEC fractions. The FIC tests were performed identically to the MIC test method outlined above. The FIC index was determined as previously described^30^. Equations (1) and (2) was used to determine the FIC values for cefoxitin or each bioactive material, and Eq. (3) was used to calculate the FIC index to determine the combinatory effect of the two components.

1 $$FIC_{A } = \frac{{MIC_{C} }}{{MIC_{A} }}$$

2 $$FIC_{B } = \frac{{MIC_{C} }}{{MIC_{B} }}$$

3 $$FIC_{i} = \sum FIC = FIC_{A} + FIC_{B}$$

where FIC_A_ and FIC_B_ are the FIC values for drug A and drug B, respectively. MIC_A_ and MIC_B_ are the respective MICs of drug A and drug B alone. MIC_C_ is the MIC of drug A and drug B in combination. The FIC index (FIC_i_) is the sum of FIC_A_ and FIC_B_.

### MRSA culture growth

For the following physiological and qPCR assays, each respective MRSA strain was grown as follows. Each MRSA strain was inoculated into 10 mL of cation-adjusted Mueller Hinton media (“untreated”) and 10 mL of cation-adjusted Mueller Hinton media supplemented with 30 mg/mL of probiotic bioactive material (“treated”) then incubated at 37 ± 1 °C and 200 rpm shaking for 24 h unless otherwise stated.

### Carotenoid measurements

The samples were prepared as described above. Following incubation, *Staphylococcal* carotenoids were extracted using a previously established methanol extraction protocol^23^. The supernatant containing the extracted carotenoids was pipetted in 200 µL (with duplicates) into a standard 96-well plate and the absorbance at a wavelength of 450 nm (A450) was measured with a SpectraMax M2 microplate reader (Molecular Devices, San Jose, USA).

### Oxidant susceptibility assays

The samples were prepared as described above. Following incubation, samples were centrifuged at 4000 rpm for 15 min, washed in 1× Dulbecco’s PBS solution (VWR Life Science, Radnor, USA), and resuspended at approximately 10^9^–10^10^ CFU/mL in 1.5% v/v hydrogen peroxide solution. Oxidant susceptibility assays were then performed following a previously established protocol^23^.

### Blood agar growth testing

The samples were prepared as described. Serial dilutions of the samples were performed and 100 µL of diluted sample was plated in duplicate on TSA plates supplemented with 5% v/v sterile defibrinated sheep blood (Cedarlane, Burlington, Ontario, Canada). Plates were incubated for 15–20 h at 37 ± 1 °C and analyzed for hemolysis and CFU/mL counts.

### RNA extraction, cDNA synthesis and qPCR testing

The samples were prepared as described above, and then incubated at 37 ± 1 °C and 200 rpm shaking for 4 h and 6 h for MRSA 414M samples and 5 h and 6 h for MRSA 81M samples. RNA extractions of the samples were performed according to the illustra™ RNAspin kit (GE Healthcare, Buckinghamshire, UK). Reverse transcriptions and cDNA syntheses were performed according to the High Capacity cDNA Reverse Transcription kit (Applied Biosystems, Foster City, USA). qPCR experiments were performed using a CFX96™ Real-Time System C1000™ Thermal Cycler and data analyses were performed with the accompanying CFX Manager 3.1 software system (Bio-Rad, Hercules, USA). The selected genes and their primer pairs are listed in Supplementary Table S1.

### Scanning electron microscopy and light microscopy

The samples were prepared as described above, and then incubated at 37 ± 1 °C and 200 rpm shaking for 4 h, 6 h and 24 h for both 414M and 81M samples. Following incubation, samples were stained with Crystal Violet, prepared for light microscopy and observed using the Upright Revolve 4 Microscope equipped with an iPad Pro (ECHO Inc., San Diego, USA). SEM samples were prepared as previously described^44^. The samples were mounted on stubs using double sided tape and sputter-coated with gold using/by a Denton Vacuum system and observed with a Hitachi TM3000 Scanning Electron Microscope (Hitachi High-Technologies Corporation, Tokyo, Japan) operated at 15 kV.

### Statistical methods

MIC data were analyzed by two-way ANOVA followed by Dunnett’s multiple comparisons test using GraphPad Prism version 9 (GraphPad software, San Diego, California). All qPCR data were analyzed by ANOVA using CFX Manager 3.1 software system (Bio-Rad, Hercules, USA). Data for carotenoid absorption assay and SCV percent cell counts were analyzed by Mann–Whitney U statistical testing using Microsoft Excel. Hydrogen-peroxide cell killing data were analyzed by Wilcoxon signed-rank for paired statistical testing using Microsoft Excel. All stated replicates (n) are the average biological value of technical duplicates.

## Supplementary Information

Supplementary Information.

## Data Availability

The datasets generated during and/or analyzed during the current study are available from the corresponding author upon request.
