# Supplementary material for: Probiotic disruption of quorum sensing reduces virulence and increases cefoxitin sensitivity in methicillin-resistant Staphylococcus aureus
Source: Sci Rep. 2023 Mar 16;13:4373. doi: 10.1038/s41598-023-31474-2 (PMC10020441; doi:10.1038/s41598-023-31474-2)
Supplement: Supplementary file 1 — Supplementary Information. [file 41598_2023_31474_MOESM1_ESM.docx]

**Supplementary Information**

**Probiotic disruption of quorum sensing reduces virulence and increases cefoxitin sensitivity in methicillin resistant Staphylococcus aureus**

Monica Angela Cella^1^, Thomas Coulson^2^*, Samantha MacEachern^2^, Sara Badr^1^, Ali Ahmadi^1^, Mahdis Sadat Tabatabaei^2^, Alain Labbe^2^* and Mansel William Griffiths^3,4^

*Corresponding author

^1^Department of Mechanical Engineering, École de Technologie Supérieure (ÉTS), Montreal, QC, Canada, H3C 1K3

^2^MicroSintesis Inc., Victoria, Prince Edward Island, Canada, COA 2G0

^3^Canadian Research Institute for Food Safety, University of Guelph, Guelph, Ontario, Canada, N1G 2W1

^4^Food Science Department, University of Guelph, Guelph, Ontario, Canad,a N1G 2W1

**Supplementary Table S1.** Target genes and the respective primers used in RT-qPCR. Thermocycling conditions for all primers were as follows: 50˚C for 2 min, 95˚C for 2 min, 40 cycles of 95˚C for 15 sec, 49˚C for 35 sec, 72˚C for 45 sec.

| Gene | Forward primer (5’-3’) | Reverse primer (5’-3’) | Reference |
| --- | --- | --- | --- |
| 16s | ACGTGGATAACCTACCTATAAGACTGGGAT | TACCTTACCAACTAGCTAATGCAGCG | [1] |
| agrA | GTGAAATTCGTAAGCATGACCCAGTTG | TGTAAGCGTGTATGTGCAGTTTCTAAAC | [2] |
| asp23 | CAAGAACAAAATCAAGAGCCTCAAT | CTTCACGTGCAGCGATACCA | [3] |
| crtM | CAATGTTTGAAACGGACGCTG | CGATTCACCAAGTCTTCTTGCG | [4] |
| hla | TAATGAATCCTGTCGCTAATGCC | CACCTGTTTTTACTGTAGTATTGCTTCC | [5] |
| luxS | GGTTGCCAAACTGGTTTCTAT | CCCAGCCACATTGTACTTCA | This study |


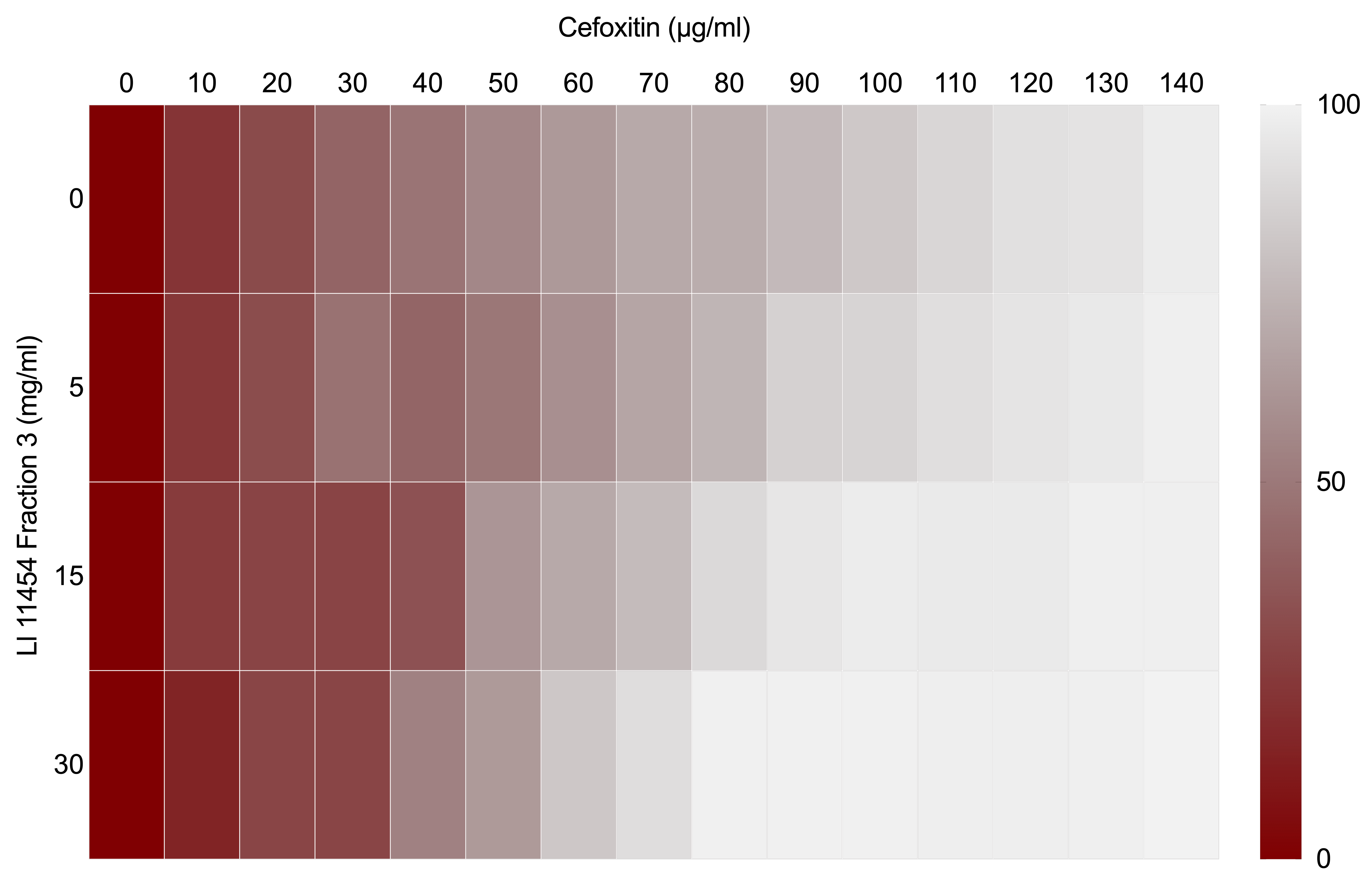

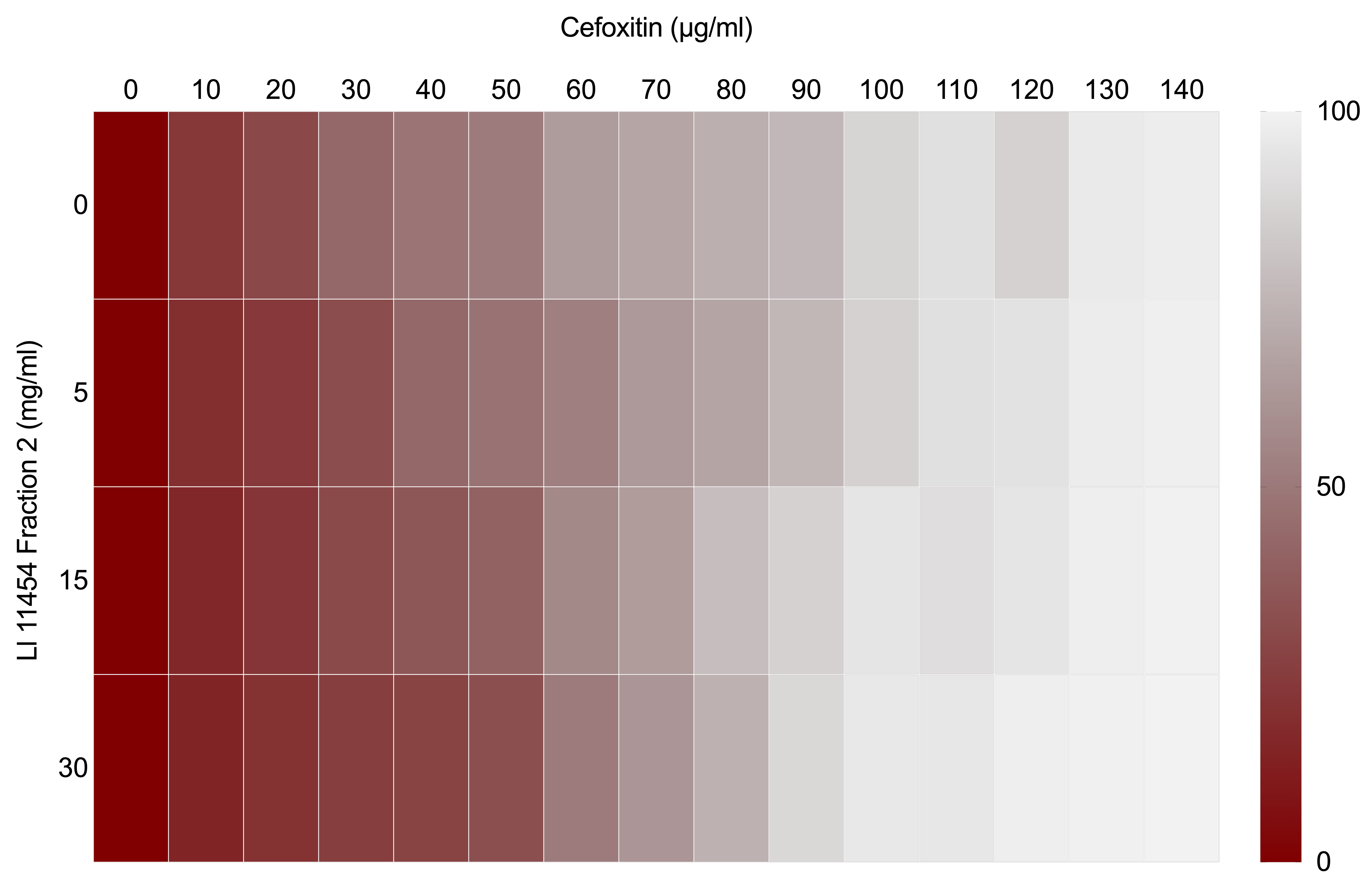

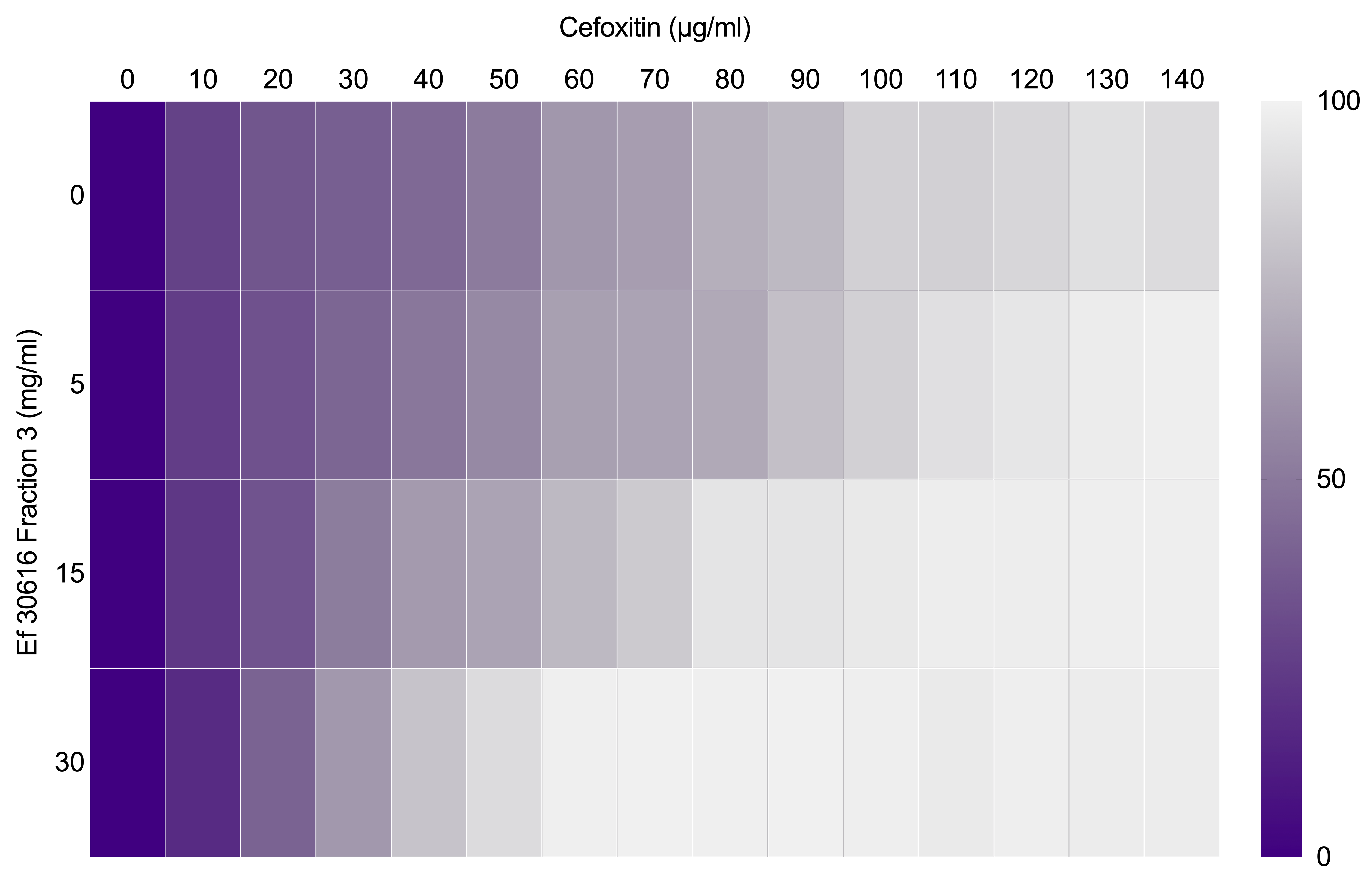

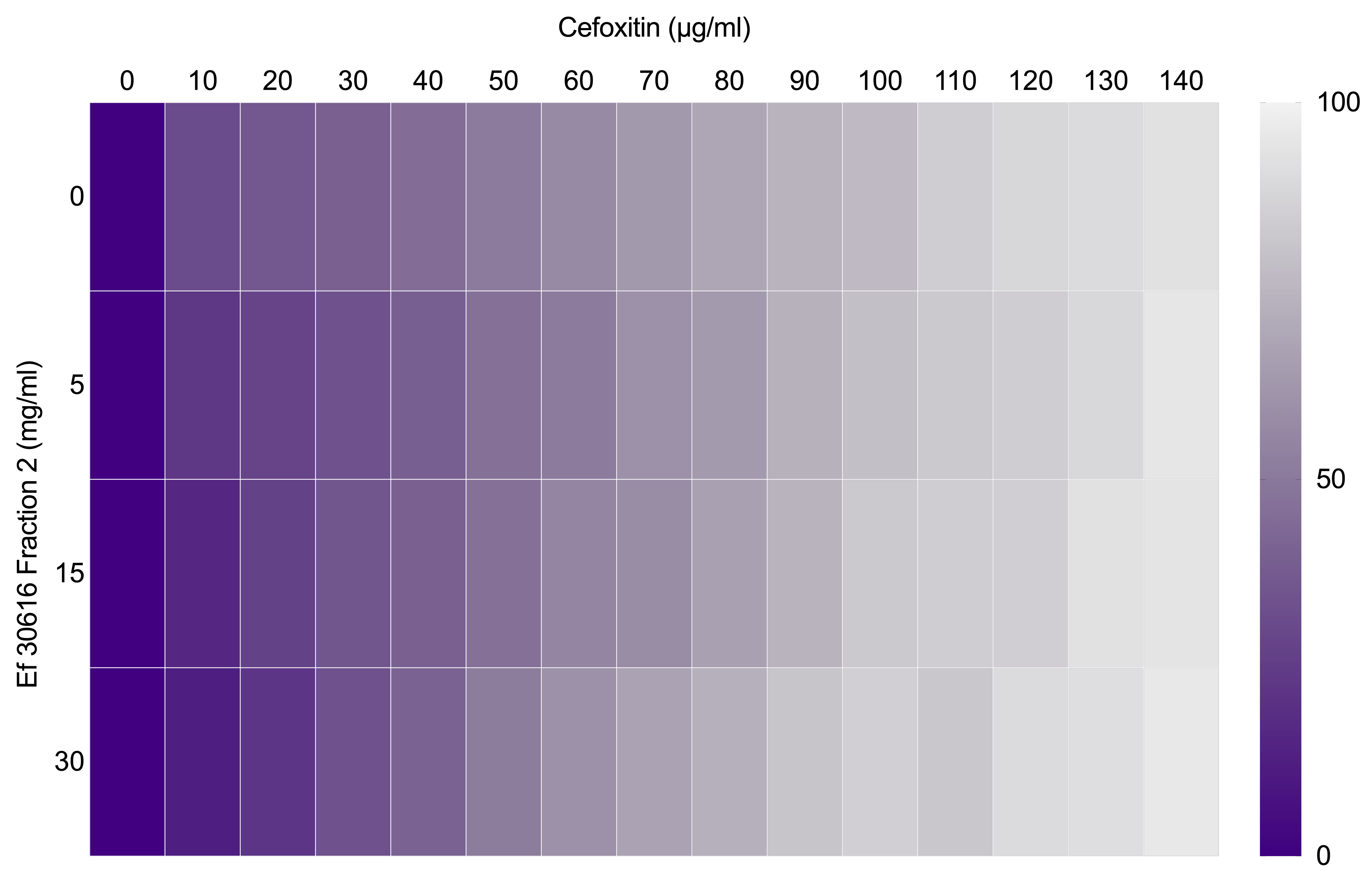

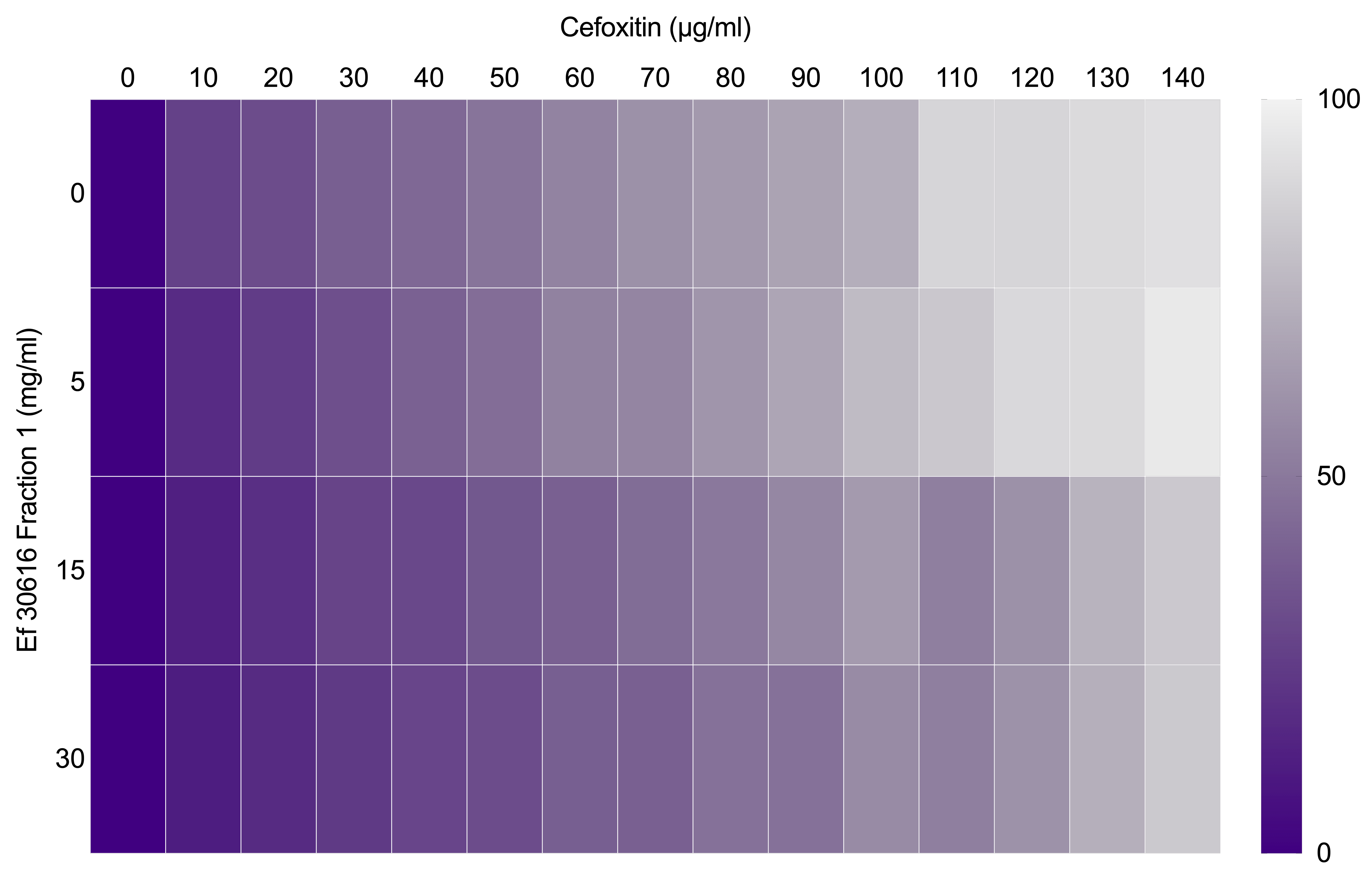

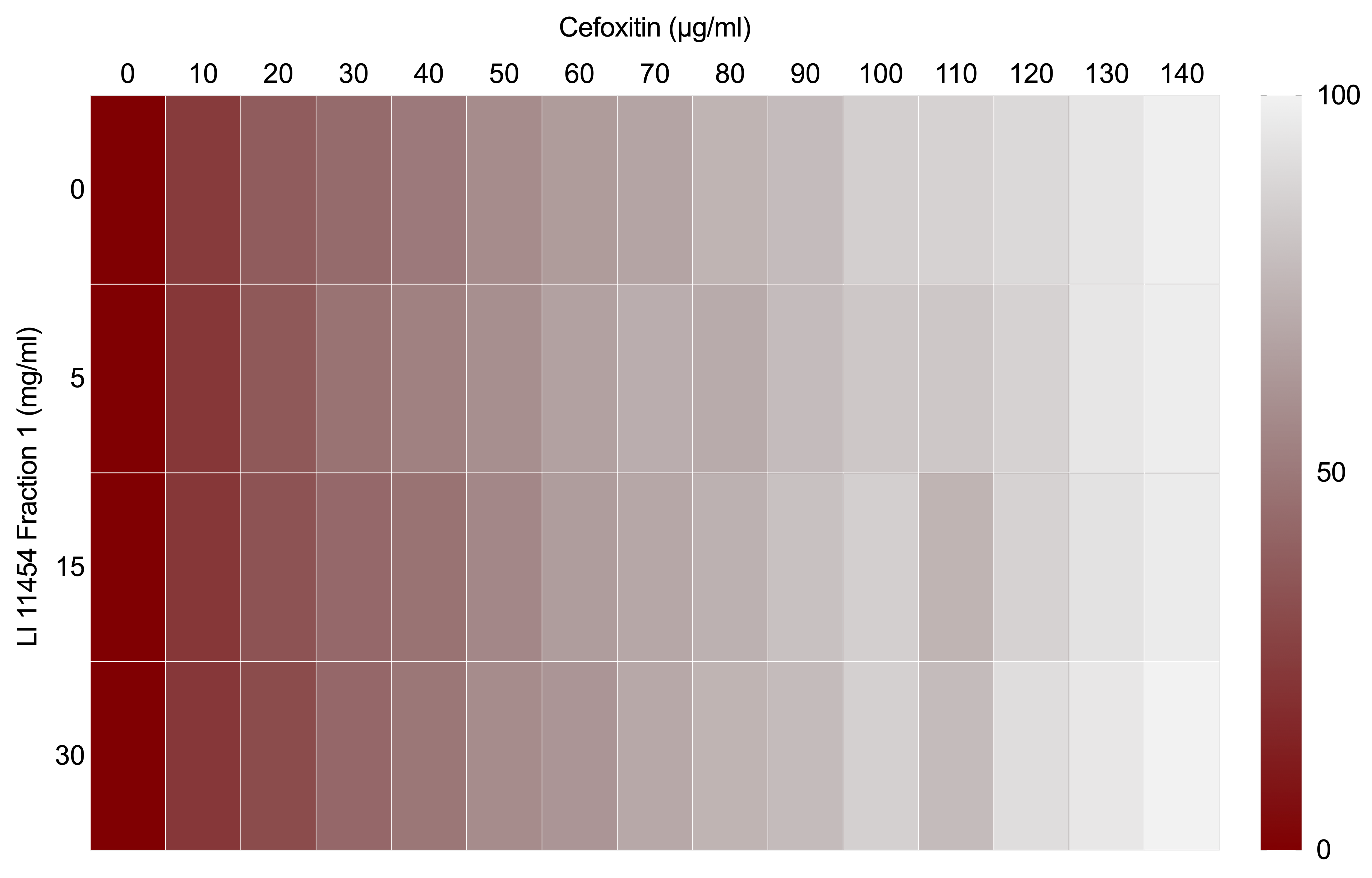


f

e

d

c

b

a

**Supplementary Figure S1.** Heat plots of MIC percent growth inhibition of MRSA 81M in combination with cefoxitin (0-140 µg/ml) and SEC fractions one, two and three from (a-c) Ef 30616 or (b-f) Ll 11454 at 5, 15 and 30 mg/ml of equivalent bulk material. Averages of biological replicates are shown for heat maps and are presented as the means in terms of percent growth inhibition (ANOVA, p < 0.05 Dunnett’s multiple comparison test).


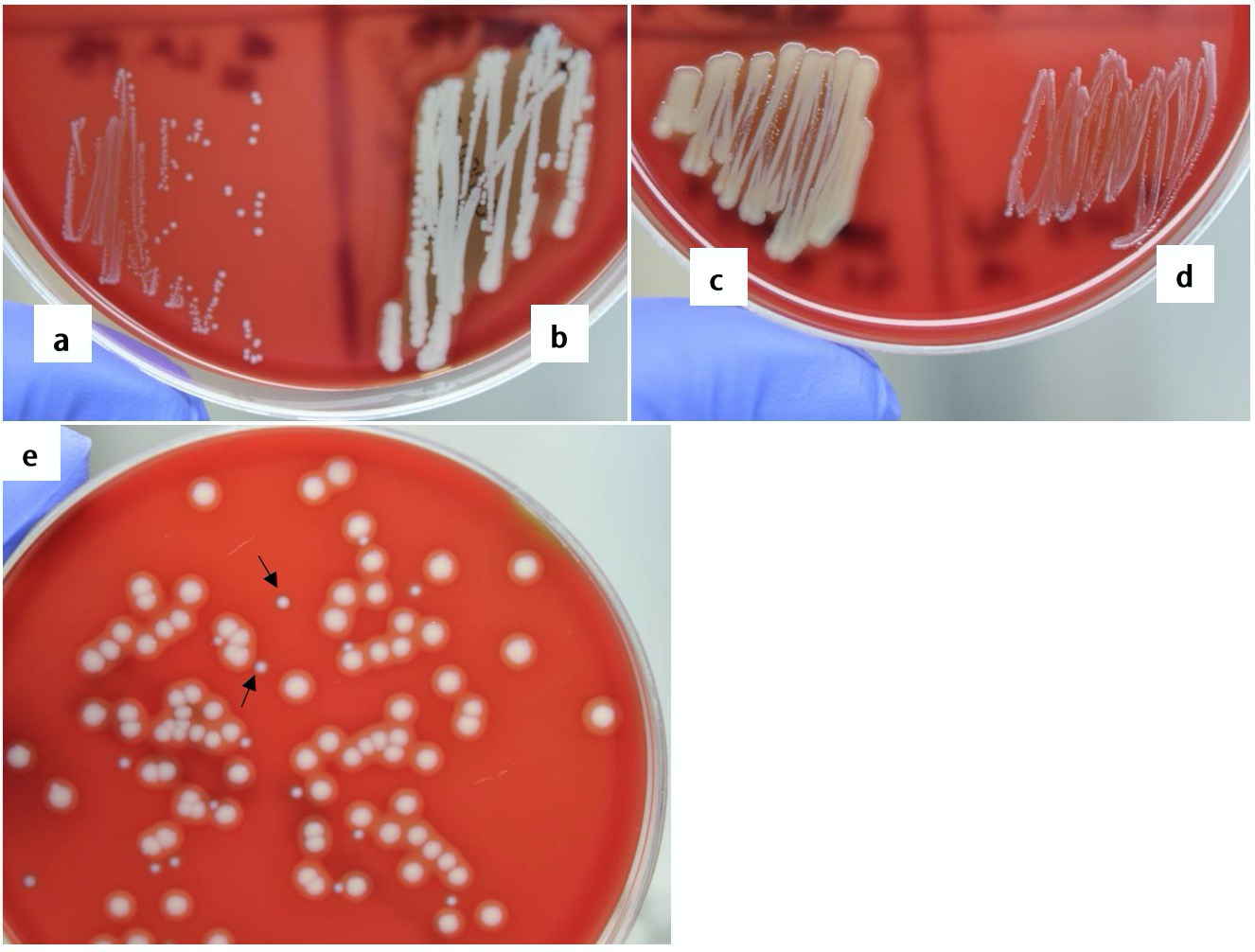


**Supplementary Figure S2**. Transfer streak plates from original parent colonies following 24 hr of incubation at 37˚C ± 1˚C: (a) 30 mg/mL Ef 30616 CFSM-treated MRSA 414M, (b) untreated MRSA 414M, (c) untreated MRSA 81M and (d) 30 mg/mL Ef 30616 CFSM-treated MRSA 81M. (e) Alpha-hemolysis rings of 30 mg/mL Ef 30616 CFSM-treated MRSA 414M. Arrows indicate MRSA 414M SCVs lacking hemolysis rings.

**b**

**a**


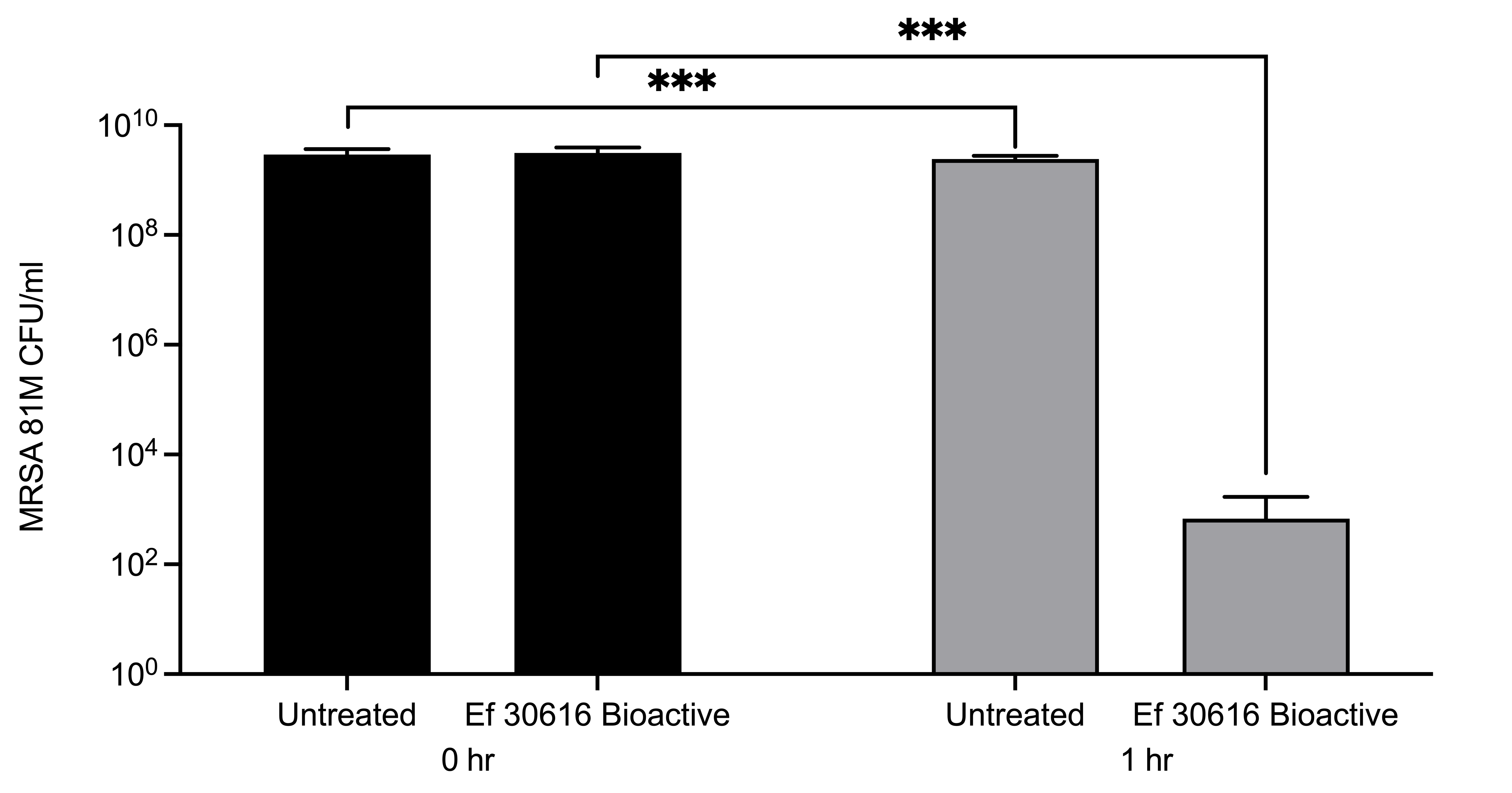

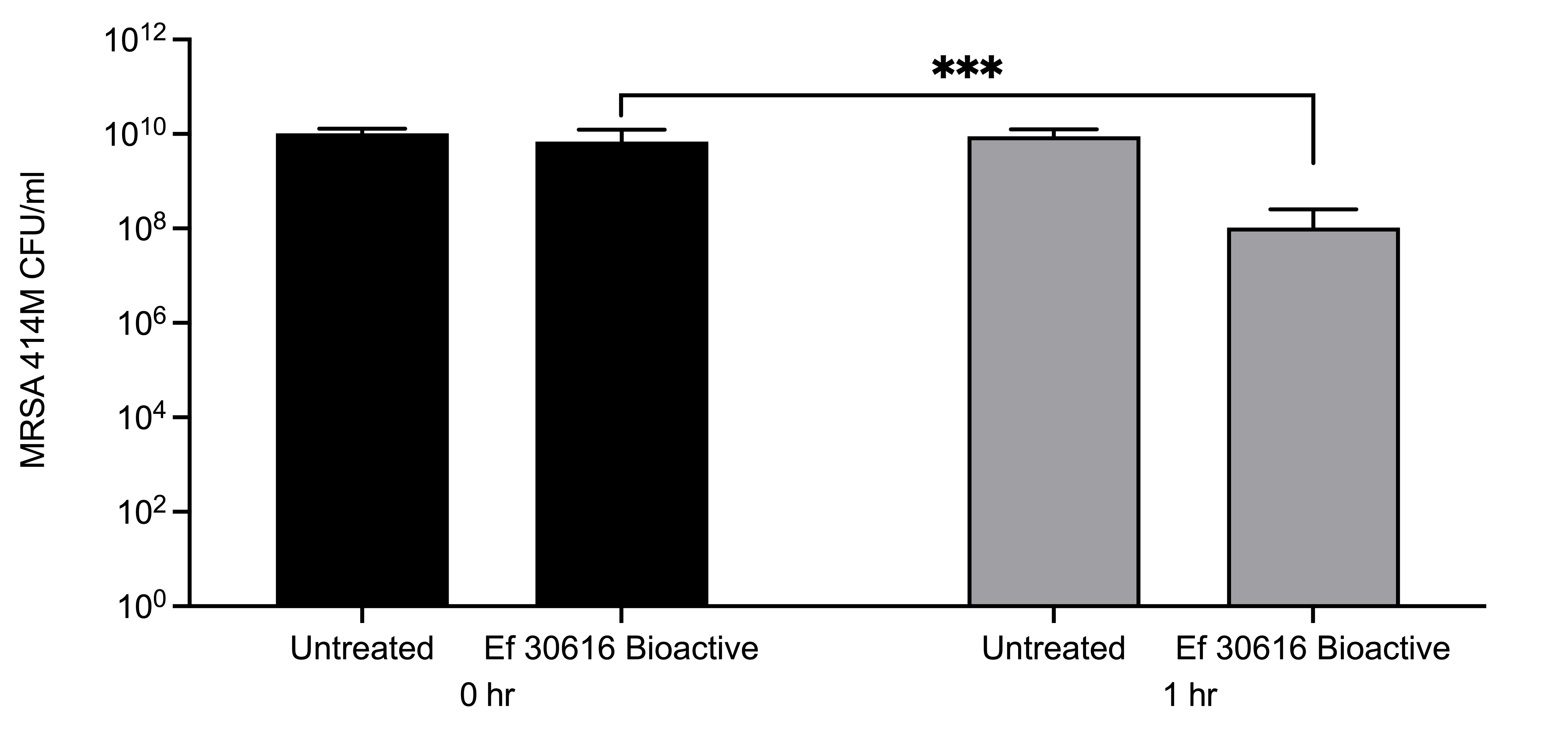


**Supplementary Figure S3**. Decreased Staphylococcus survival when treated with 1.5% v/v hydrogen peroxide for (a) MRSA 81M (n=3) and (b) MRSA 414M (n=3) before and after 1 hr incubations at 37˚C ± 1˚C in 1xPBS solution without and with 30 mg/mL Ef 30616 CFSM. The starting cell concentrations (T=0hr) and surviving cell concentrations (T=1hr) are shown as the average CFU/mL. All biological replicates were performed in technical duplicate. Following incubation with 1.5% v/v hydrogen peroxide, both bioactive treated MRSA 81M and 414M had significant levels of cell death at >99.99% and 99.67%, respectively (W=0; ***, p<0.001, Wilcoxon signed-rank). Untreated MRSA 81M also had a small, yet significant, level of cell death (19.70%) following incubation with bioactive material (W=0; ***, p<0.001, Wilcoxon signed-rank). MRSA 414M also had a small amount of cell death (16.67%), however this was not significant (W=5; p>0.05, Wilcoxon signed-rank).


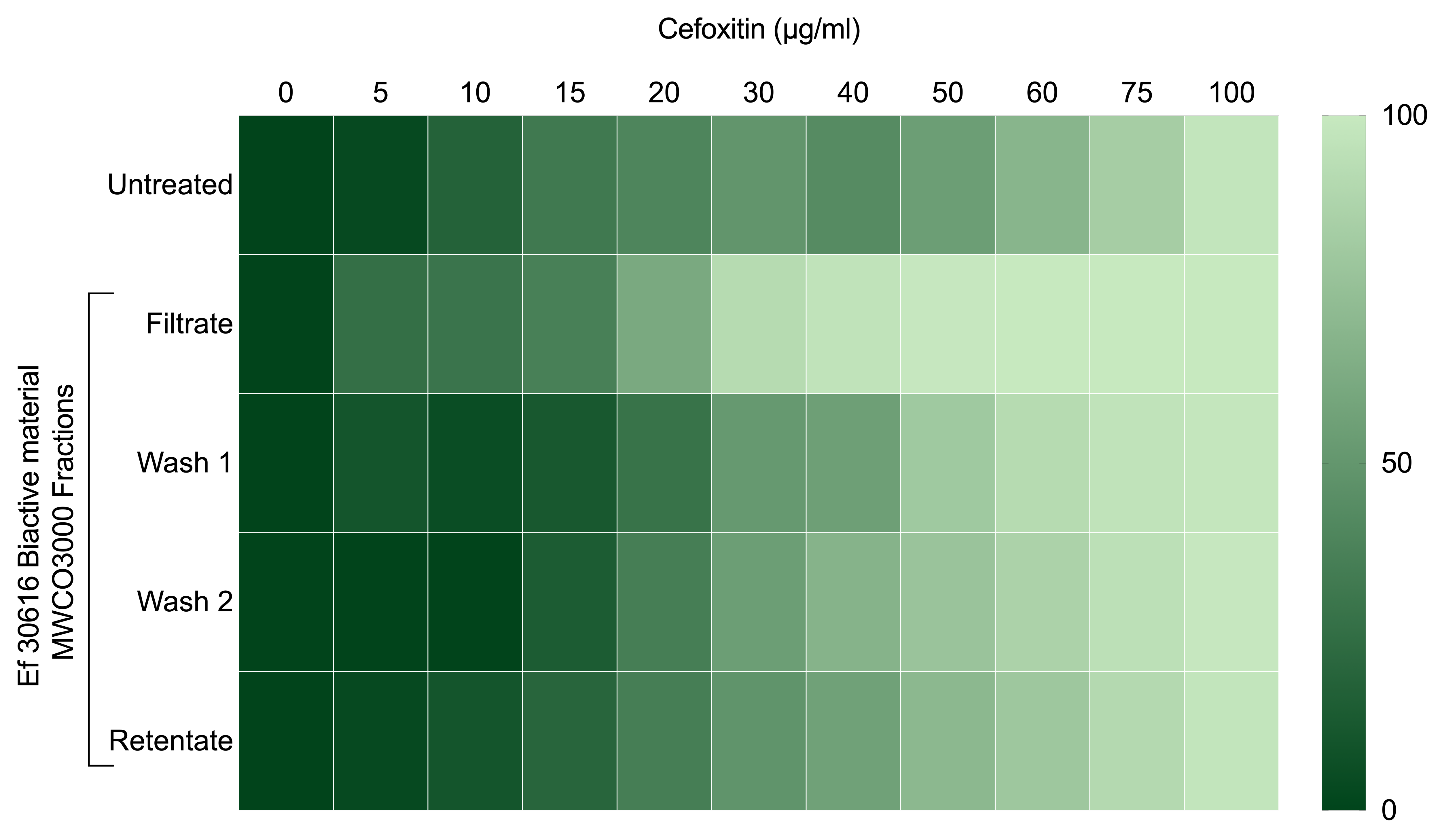


**Supplementary Figure S4.** Heat plot of the MIC percent growth inhibition of MRSA 81M by combination testing of the antibiotic cefoxitin (0-100 µg/mL) and the Ef 30616 bioactive containing CFSM material (30 mg/mL) MWCO 3000 Da filtrate (i.e. <3000 Da), two washes of the retentate pellet, and retentate only (i.e. >3000Da) (n=3). All biological replicates were performed in technical duplicates. The 30 mg/mL bioactive material concentration was selected for fractional testing as it was the lowest tested concentration resulting in synergistic FIC index values for an MRSA clinical strain. The two washes of the retentate that were performed also showed some residual biological activity.

**References**

1. Xiu, J. *et al.* Platelets directly regulate DNA damage and division of Staphylococcus aureus. *FASEB J* **32**, 3707-3716 (2018).

2. Baroja, M. L., Herfst, C. A., Kasper, K. J., Xu, S. X., Gillett, D. A., Li, J., Reid, G. & McCormick, J. L. The SaeRS Two-Component System Is a Direct and Dominant Transcriptional Activator of Toxic Shock Syndrome Toxin 1 in Staphylococcus aureus. *J Bacteriol* **19**, 2732-2742 (2016).

3. Chatterjee, I., Becker, P., Grundmeier, M., Bischoff, M., Somerville, G. A., Peters, G., Sinha, B., Harraghy, N., Proctor, R. A., & Herrmann, M. Staphylococcus aureus ClpC Is Required for Stress Resistance, Aconitase Activity, Growth Recovery, and Death.  *J Bacteriol* **187**, 4488-4496 (2005).

4. Tiwari, K. B., Gatto, C & Wilkinson, B. J. Interrelationships among Fatty Acid Composition, Staphyloxanthin Content, Fluidity, and Carbon Flow in the Staphylococcus aureus Membrane. *Molecules* **23**, 1201 (2018).

5. Tavares, A., Nielson, J. B., Boye, K., Rohde, S., Paulo, A. C., Westh, H., Schønning, K., de Lencastre, H & Miragaia, M. Insights into Alpha-Hemolysin (Hla) Evolution and Expression among Staphylococcus aureus Clones with Hospital and Community Origin. *PLoS One* **9**, e98635 (2014).
